# Synthesis of 2-Nitro-2,3-Unsaturated Glycosides by a Nanomagnetic Catalyst Fe_3_O_4_@C@Fe(III)

**DOI:** 10.3389/fchem.2022.865012

**Published:** 2022-05-11

**Authors:** Yu Yang, Nan Jiang, Yuling Mei, Zekun Ding, Jianbo Zhang

**Affiliations:** School of Chemistry and Molecular Engineering, East China Normal University, Shanghai, China

**Keywords:** 2-nitro glycals, Ferrier rearrangement, nanomagnetic catalyst, Fe_3_O_4_@C@Fe(III), recyclable, 2-nitro-2, 3 unsaturated glycosides

## Abstract

A sustainable magnetic core-shell nanocatalyst Fe_3_O_4_@C@Fe(III) was successfully applied in the synthesis of a series of 2-nitro-2,3-unsaturated *O*-glycosides with excellent yields (up to 89%) and high stereoselectivity (α:β > 19:1). The substrate ranges are widely applicable, including different kinds of alcohols and even structurally complex acceptors. In addition, phenols could be applied in good yields. Moreover, the catalyst could be easily separated from the reaction by the application of an external magnetic force and reused a minimum of five times without any significant decrease in catalytic performance.

## Introduction

**FIGURE 1:**
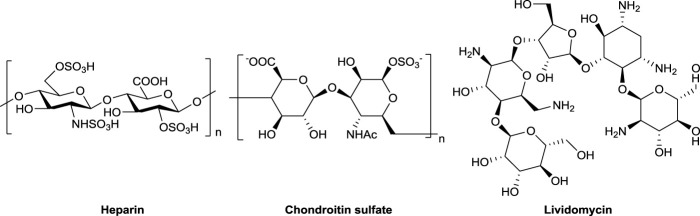
Representative 2-amino-2-deoxyglycoside–containing natural products.

Amino deoxy sugars are commonly found in bioactive natural products (Wang et al., 2013; Dalziel et al., 2014; Canales et al., 2017; Wild et al., 2018; Shariatinia, 2019; Zhang et al., 2020). In particular, one of the most important amino deoxy sugars, 2-amino-2-deoxy glucose, is an essential unit of glucosaminoglycans (such as heparin and chondroitin sulfate) and many antibiotics (such as lividomycin), as shown in Figure 1. However, 2-amino-2-deoxy sugar–containing pharmaceutical agents with high purity and single stereo configuration are difficult to be extracted from natural sources due to their limited inventories and biosynthetic microheterogeneity (Suzuki et al., 2021; Zeng et al., 2021). Therefore, scientists have been making unremitting efforts to synthesize these compounds through chemical methods (Iglesias-Guerra et al., 1999; Vega-Pérez et al., 1999; Sugita et al., 2014).

**SCHEME 1:**
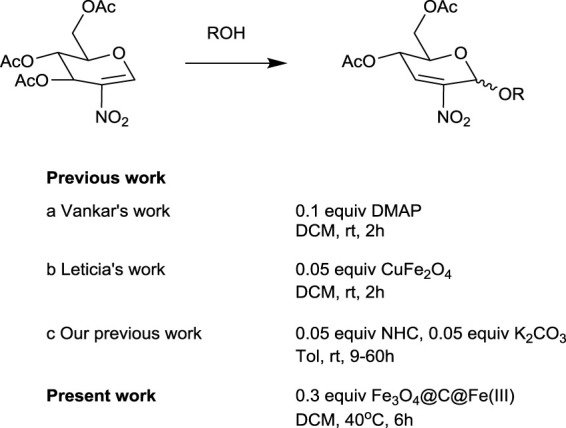
Previous work and present work.

2-nitro-2,3 unsaturated glycosides are a kind of important intermediates for the synthesis of 2-amino-2-deoxy sugars. However, so far, there are few reports illustrating efficient ways to obtain these compounds (Scheme 1). In 2013, Dharuman et al. (2013) initially reported their exploration on Ferrier rearrangement of 2-nitro glycals. 4-dimethylaminopyridine, an organic base, was used to successfully catalyze the reaction. However, the number of substrates suitable for this reaction condition was only five, and the yield was generally not very high (57–74%), probably due to the occurrence of deacylation side reaction under alkaline condition. In 2018, Lafuente et al. (2019) promoted the Ferrier rearrangement of 2-nitro glycals with several *O*-nucleophiles in the presence of CuFe_2_O_4_. The catalyst could be separated by an external magnet, while the reaction products were obtained in good yields and high stereoselectivity. However, the preparation process of the Cu–Fe spinel catalyst was complicated, and the conditions were very harsh, so the number of suitable substrates was only eight. Recently, we published an article in which N-heterocyclic carbenes (NHCs) catalyzed Ferrier rearrangement smoothly with the aid of potassium carbonate (Jiang et al., 2021). The reaction conditions were mild, and the range of alcoholic substrates was wide, but the reaction was time-consuming and suffered deacylation as well. On the basis of reports in the literature (Michigami and Hayashi, 2012; Chen and Lin, 2013; Chen et al., 2017; Dong et al., 2019), we concluded that the nitro substituent at the C-2 position has a remarkable effect in the sugar ring and that electron-withdrawing groups reduce the electron density of the oxocarbenium intermediate in the normal Ferrier rearrangement. Consequently, the development of a green and broadly adaptive protocol for the synthesis of 2-nitro-2,3-unsaturated glycosides remains a challenge.

Our research group has been conducting a series of explorations on solid acid catalysts in environmentally benign reactions, such as H_2_SO_4_–SiO_2_, FeCl_3_·6H_2_O/C, and FeCl_3_/C, which have been applied in Ferrier rearrangement reactions with good yield in short time (Zhang et al., 2013; Zhang et al., 2018; Mei et al., 2020). Furthermore, these heterogenous catalysts could usually be easily separated from the reaction media and recycled to enhance their applicability. In addition, we are introducing a nano-magnetic core-shell catalyst into a Ferrier rearrangement of glycals due to their extraordinary noncontact magnetic separation (Wang et al., 2019; Zhang et al., 2021). Previously, Fe_3_O_4_@C-SO_3_H and Fe_3_O_4_@C@Fe(Ⅲ) have been studied for the synthesis of *O*-2,3-unsaturated glycosides (Zhang et al., 2017; Dong et al., 2019; Jiang et al., 2020a). The donors included 3,4,6-tri-*O*-acetyl-glucal, 3,4,6-tri-*O*-acetyl-2-haloglucals, and 3,4-di-*O*-acetyl-L-rhamnal. The acceptors consisted of primary alcohols, secondary alcohols, tert-butanol, unsaturated alcohols, halogenated alcohol, sterol, sugars, and phenols. O-2,3-unsaturated glycosides were obtained rapidly (<3 h) and efficiently (up to 98%) in good α-selectivity (α:β *>* 5:1 to 19:1). Moreover, the catalyst could be easily separated from the reaction with an external magnetic force and reused several times without any significant decrease in the yields of the products after every recycle, suggesting it to be a promising green catalyst in 2,3-unsaturated glycosides syntheses. Therefore, we would like to continue the research of Fe_3_O_4_@C@Fe(III) in the synthesis of 2-nitro unsaturated glycosides, which has not been attempted before.

## Materials and Methods

### General Information

All reactions were carried out under a dry nitrogen atmosphere. All solvents and reagents were obtained from commercial sources, unless otherwise stated, and were purified according to standard procedures. The removal of the solvent *in vacuo* refers to distillation using a rotary evaporator attached to an efficient vacuum pump. ^1^H NMR and ^13^C NMR spectra were recorded on a Bruker DRX-500 NMR spectrometer in solutions of CDCl_3_ using tetramethylsilane as the internal standard. Mass spectra were determined on LTQ-XL (Thermo Scientific, United States) with an (ESI) ion trap mass spectrometer.

### General Procedure for the Synthesis of 2-Nitro-2,3-Unsaturated Glycosides

To a mixture of 2-nitroglycal (63.4 mg, 0.2 mmol), acceptor (0.24 mmol), and the nanomagnetic catalyst Fe_3_O_4_@C@Fe(III) (0.06 mmol) was added DCM (2.0 ml) under a nitrogen atmosphere. The reaction mixture was stirred at 40°C and monitored by TLC (PE/EA, 2:1) until the reactant was consumed completely. After completion of the reaction, the catalyst was separated from the reaction with an external magnetic force and washed with dichloromethane. The solvent was removed under reduced pressure to afford a crude product, which was purified by silica gel flash chromatography with a solvent system (PE/EA, 4:1) to yield 2-nitro-2,3-unsaturated glycosides.

### Experimental Data

[(2R,3S,6S)-3-acetoxy-6-ethoxy-5-nitro-3,6-dihydro-2H-pyran-2-yl] methyl acetate (**3a**): colorless syrup. ^1^H NMR (500 MHz, CDCl_3_) δ 7.16 (d, J = 1.0 Hz, 1H), 5.57 (d, J = 6.7 Hz, 2H), 4.25 (s, 3H), 3.89 (ddd, J = 15.7, 8.3, 4.0 Hz, 1H), 3.76 (ddd, J = 8.7, 7.5, 4.8 Hz, 1H), 2.14 (s, 3H), 2.10 (s, 3H), 1.26 (t, J = 7.1 Hz, 3H). ^13^C NMR (125 MHz, CDCl_3_) δ 170.70, 169.91, 148.44, 132.50, 92.30, 66.41, 65.90, 64.59, 62.16, 20.82, 20.82, 15.15. HRMS (ESI): m/z calculated for C_12_H_17_NO_8_Na [M + Na]^+^326.0846, found 326.0849.

[(2R,3S,6S)-3-acetoxy-6-methoxy-5-nitro-3,6-dihydro-2H-pyran-2-yl] methyl acetate (**3b**): colorless syrup. ^1^H NMR (500 MHz, CDCl_3_) δ 7.19 (d, J = 2.2 Hz, 1H), 5.58 (d, J = 6.7 Hz, 1H), 5.48 (s, 1H), 4.31–4.26 (m, 2H), 4.25–4.20 (m, 1H), 3.56 (s, 3H), 2.15 (s, 3H), 2.12 (s, 3H). HRMS (ESI): m/z calculated for C_11_H_15_NO_8_Na [M + Na]^+^312.0690, found 312.0694.

[(2R,3S,6S)-3-acetoxy-6-(hexyloxy)-5-nitro-3,6-dihydro-2H-pyran-2-yl] methyl acetate (**3c**): colorless syrup. ^1^H NMR (500 MHz, CDCl_3_) δ 7.16 (d, J = 1.6 Hz, 1H), 5.57 (d, J = 6.7 Hz, 2H), 4.30–4.20 (m, 3H), 3.85–3.78 (m, 1H), 3.69–3.63 (m, 1H), 2.14 (s, 3H), 2.10 (s, 3H), 1.31–1.25 (m, 11H), 0.87 (t, J = 6.8 Hz, 4H). ^13^C NMR (125 MHz, CDCl_3_) δ 170.70, 169.91, 148.41, 132.48, 92.48, 70.50, 66.45, 64.58, 62.17, 31.94, 29.54, 29.40, 29.34, 26.16, 22.77, 20.83, 14.21. HRMS (ESI): m/z calculated for C_18_H_29_NO_8_Na [M + Na]^+^410.1785, found 410.1797.

[(2R,3S,6S)-3-acetoxy-6-(benzyloxy)-5-nitro-3,6-dihydro-2H-pyran-2-yl] methyl acetate (**3d**): colorless syrup. ^1^H NMR (500 MHz, CDCl_3_) δ 7.38–7.33 (m, 5H), 7.20 (d, J = 2.1 Hz, 1H), 5.71 (s, 1H), 5.59 (dd, J = 9.4, 1.4 Hz, 1H), 4.84 (d, J = 11.3 Hz, 1H), 4.76 (d, J = 11.3 Hz, 1H), 4.27 (ddd, J = 19.5, 9.8, 3.4 Hz, 2H), 4.12 (q, J = 7.1 Hz, 1H), 2.13 (s, 3H), 2.11 (s, 3H). HRMS (ESI): m/z calculated for C_17_H_19_NO_8_Na [M + Na]^+^388.1003, found 388.1013.

[(2R,3S,6S)-3-acetoxy-6-(allyloxy)-5-nitro-3,6-dihydro-2H-pyran-2-yl] methyl acetate(**3e**): colorless syrup. ^1^H NMR (500 MHz, CDCl_3_) δ 7.19 (d, J = 2.1 Hz, 1H), 5.95 (ddt, J = 16.7, 10.5, 5.9 Hz, 1H), 5.64 (s, 1H), 5.60–5.55 (m, 1H), 5.34 (dd, J = 17.2, 1.3 Hz, 1H), 5.30–5.25 (m, 1H), 4.32 (dd, J = 12.5, 5.5 Hz, 1H), 4.27 (d, J = 8.7 Hz, 3H), 4.24–4.20 (m, 1H), 2.15 (s, 3H), 2.11 (s, 3H). HRMS (ESI): m/z calculated for C_13_H_17_NO_8_Na [M + Na]^+^338.0846, found 338.0855.

[(2R,3S,6S)-3-acetoxy-5-nitro-6-(2,2,2-trichloroethoxy)-3,6-dihydro-2H-pyran-2-yl] methyl acetate (**3f**): colorless syrup. ^1^H NMR (500 MHz, CDCl_3_) δ 7.30 (d, J = 1.6 Hz, 1H), 5.79 (s, 1H), 5.62 (d, J = 9.8 Hz, 1H), 4.38 (d, J = 3.8 Hz, 2H), 4.28 (qd, J = 12.3, 3.5 Hz, 3H), 2.17 (s, 3H), 2.10 (s, 3H). ^13^C NMR (125 MHz, CDCl_3_) δ 170.62, 169.85, 146.95, 133.97, 95.43, 92.23, 80.72, 67.43, 64.31, 61.81, 20.82. HRMS (ESI): m/z calculated for C_12_H_14_Cl_3_NO_8_Na [M + Na]^+^427.9677, found 427.9686.

{(2R,3S,6S)-3-acetoxy-6-[(5-formylfuran-2-yl) methoxy]-5-nitro-3,6-dihydro-2H-pyran-2-yl} methyl acetate (**3g**): colorless syrup. ^1^H NMR (500 MHz, CDCl_3_) δ 9.64 (s, 1H), 7.23 (s, 2H), 6.61 (d, J = 2.8 Hz, 1H), 5.71 (s, 1H), 5.60 (d, J = 9.2 Hz, 1H), 4.84 (q, J = 13.3 Hz, 2H), 4.26 (dd, J = 11.8, 8.0 Hz, 3H), 2.15 (s, 3H), 2.11 (s, 3H). ^13^C NMR (125 MHz, CDCl_3_) δ 177.86, 170.64, 169.86, 156.42, 153.05, 147.51, 133.59, 121.95, 112.26, 100.14, 66.89, 64.37, 63.32, 61.82, 20.84, 20.82. HRMS (ESI): m/z calculated for C_16_H_17_NO_10_Na [M + Na] ^+^406.0745, found 406.0746.

((2R,3S,6S)-3-acetoxy-5-nitro-6-{[(2R,3R,5R,6S)-3,4,5-tris(benzyloxy)-6-methoxytetrahydro-2H-pyran-2-yl] methoxy}-3,6-dihydro-2H-pyran-2-yl) methyl acetate (**3h**): colorless syrup. ^1^H NMR (500 MHz, CDCl_3_) δ 7.39–7.26 (m, 15H), 7.17 (d, J = 1.3 Hz, 1H), 5.73 (s, 1H), 5.56 (d, J = 9.5 Hz, 1H), 4.98 (d, J = 10.9 Hz, 1H), 4.89 (d, J = 11.1 Hz, 1H), 4.80 (d, J = 11.1 Hz, 2H), 4.67 (d, J = 12.0 Hz, 1H), 4.58 (d, J = 11.2 Hz, 2H), 4.19 (s, 3H), 4.01 (t, J = 9.3 Hz, 1H), 3.95 (d, J = 11.5 Hz, 1H), 3.87 (dd, J = 11.7, 4.6 Hz, 1H), 3.78 (dd, J = 9.8, 3.5 Hz, 1H), 3.57 (dd, J = 9.6, 3.4 Hz, 1H), 3.48 (t, J = 9.4 Hz, 1H), 3.36 (s, 3H), 2.14 (s, 3H), 2.04 (s, 3H). ^13^C NMR (125 MHz, CDCl_3_) δ 170.65, 169.87, 148.27, 138.84, 138.32, 138.29, 132.83, 128.60, 128.57, 128.53, 128.27, 128.15, 128.04, 127.91, 127.89, 127.75, 98.21, 92.33, 82.03, 80.19, 77.61, 75.87, 75.02, 73.65, 70.48, 67.69, 66.38, 64.49, 61.98, 55.41, 20.84, 20.80. HRMS (ESI): m/z calculated for C_38_H_43_NO_13_Na [M + Na]^+^744.2627, found 744.2623.

[(2R,3S,6S)-3-acetoxy-6-isopropoxy-5-nitro-3,6-dihydro-2H-pyran-2-yl] methyl acetate(**3i**): colorless syrup. ^1^H NMR (500 MHz, CDCl_3_) δ 7.15 (d, J = 2.1 Hz, 1H), 5.67 (s, 1H), 5.55 (dd, J = 9.4, 2.0 Hz, 1H), 4.32–4.27 (m, 1H), 4.27–4.23 (m, 2H), 4.07 (dd, J = 12.4, 6.2 Hz, 1H), 2.15 (s, 3H), 2.10 (s, 3H), 1.27 (d, J = 6.2 Hz, 3H), 1.24 (d, J = 6.2 Hz, 3H). ^13^C NMR (125 MHz, CDCl_3_) δ 170.71, 169.99, 132.28, 100.14, 91.31, 73.32, 66.37, 64.72, 62.32, 23.47, 21.86, 20.86, 20.83. HRMS (ESI): m/z calculated for C_13_H_19_NO_8_Na [M + Na] ^+^340.1003, found 340.1007.

((2R,3S,6S)-3-acetoxy-6-{[(1S,2S,5R)-2-isopropyl-5-methylcyclohexyl]oxy}-5-nitro-3,6-dihydro-2H-pyran-2-yl)methyl acetate (**3j**): colorless syrup. ^1^H NMR (500 MHz, CDCl_3_) δ 7.13 (d, J = 2.1 Hz, 1H), 5.70 (s, 1H), 5.54 (d, J = 9.7 Hz, 1H), 4.31 (d, J = 9.0 Hz, 1H), 4.28–4.18 (m, 2H), 3.57 (t, J = 10.2 Hz, 1H), 2.29 (d, J = 11.8 Hz, 1H), 2.14 (s, 3H), 2.11 (s, 3H), 2.07–2.00 (m, 1H), 1.64 (dd, J = 10.0, 2.7 Hz, 2H), 1.42 (d, J = 2.9 Hz, 1H), 1.21 (d, J = 10.3 Hz, 1H), 1.08–0.98 (m, 2H), 0.93 (d, J = 5.2 Hz, 3H), 0.85 (d, J = 5.8 Hz, 4H), 0.78 (d, J = 5.6 Hz, 3H). ^13^C NMR (125 MHz, CDCl_3_) δ 170.79, 169.95, 148.94, 131.89, 93.51, 82.95, 66.38, 64.63, 62.47, 48.63, 43.12, 34.33, 31.72, 25.02, 22.95, 22.59, 21.27, 20.92, 20.86, 15.72. HRMS (ESI): m/z calculated for C_20_H_31_NO_8_Na [M + Na] ^+^436.1942, found 436.1951.

((2R,3S,6S)-3-acetoxy-6-{[(3S,8S,9S,10R,13R,14S,17R)-10,13-dimethyl-17-[(R)-6-methylheptan-2-yl]-2,3,4,7,8,9,10,11,12,13,14,15,16,17-tetradecahydro-1H-cyclopenta[a]phenanthren-3-yl] oxy}-5-nitro-3,6-dihydro-2H-pyran-2-yl)methyl acetate (**3k**): white solid. ^1^H NMR (500 MHz, CDCl_3_) δ 7.16 (d, J = 2.2 Hz, 1H), 5.71 (s, 1H), 5.54 (dd, J = 9.7, 1.5 Hz, 1H), 5.37 (d, J = 5.1 Hz, 1H), 4.34–4.29 (m, 1H), 4.28–4.22 (m, 2H), 3.65 (dd, J = 11.0, 4.9 Hz, 1H), 2.38 (ddd, J = 18.4, 11.7, 9.4 Hz, 2H), 2.14 (s, 3H), 2.10 (s, 3H), 2.04–0.68 (41H, cholesteryl-H). ^13^C NMR (125 MHz, CDCl_3_) δ 170.75, 169.95, 148.74, 140.53, 132.33, 122.41, 91.20, 80.36, 66.46, 64.72, 62.37, 56.86, 56.29, 50.27, 42.47, 40.39, 39.88, 39.66, 37.13, 36.82, 36.33, 35.93, 32.09, 32.02, 28.38, 28.16, 27.98, 24.43, 23.97, 22.97, 22.71, 21.19, 20.89, 20.86, 19.49, 18.86, 12.00. HRMS (ESI): m/z calculated for C_37_H_57_NO_8_Na [M + Na] ^+^666.3976, found 666.3987.

[(2R,3S,6R)-3-acetoxy-5-nitro-6-phenoxy-3,6-dihydro-2H-pyran-2-yl] methyl acetate (**3l**): colorless syrup. ^1^H NMR (500 MHz, CDCl_3_) δ 7.35 (dd, J = 11.8, 4.2 Hz, 3H), 7.16 (s, 1H), 7.13 (dd, J = 11.5, 4.1 Hz, 2H), 6.17 (s, 1H), 5.65 (dd, J = 9.3, 1.4 Hz, 1H), 4.46–4.41 (m, 1H), 4.31 (dd, J = 12.4, 5.2 Hz, 1H), 4.26 (dd, J = 12.3, 2.1 Hz, 1H), 2.18 (s, 3H), 2.04 (s, 3H). ^13^C NMR (125 MHz, CDCl_3_) δ 170.65, 169.89, 156.99, 155.14, 147.68, 133.71, 129.86, 124.00, 118.00, 99.90, 91.92, 67.30, 64.46, 61.95, 20.85, 20.77. HRMS (ESI): m/z calculated for C_16_H_17_NO_8_Na [M + Na] ^+^374.0846, found 374.0851.

[(2R,3S,6R)-3-acetoxy-6-(4-methoxyphenoxy)-5-nitro-3,6-dihydro-2H-pyran-2-yl] methyl acetate (**3m**): colorless syrup. ^1^H NMR (500 MHz, CDCl_3_) δ7.32 (s, 1H), 7.11 (s, 2H), 6.86 (d, *J* = 17.1 Hz, 2H), 6.02 (s, 1H), 5.64 (d, *J* = 9.9 Hz, 1H), 4.46 (s, 1H), 4.30 (s, 2H), 3.79 (d, *J* = 0.9 Hz, 3H), 2.17 (s, 3H), 2.08 (s, 3H). ^13^C NMR (126 MHz, CDCl_3_) δ 170.86, 170.10, 156.52, 151.12, 147.81, 133.76, 119.93, 115.03, 93.21, 77.11, 67.35, 64.73, 62.28, 56.01, 21.05, 21.02. HRMS (ESI): m/z calculated for C_17_H_19_NO_9_Na [M + Na] ^+^404.0952, found 404.0957.

[(2R,3R,6S)-3-acetoxy-6-(benzyloxy)-5-nitro-3,6-dihydro-2H-pyran-2-yl] methyl acetate (**3o**): colorless syrup. ^1^H NMR (500 MHz, CDCl_3_) δ 7.36 (dd, *J* = 8.7, 4.1 Hz, 5H), 7.29 (d, *J* = 5.6 Hz, 1H), 5.78 (s, 1H), 5.40 (dd, *J* = 5.6, 2.8 Hz, 1H), 4.84 (d, *J* = 11.2 Hz, 1H), 4.73 (d, *J* = 11.2 Hz, 1H), 4.49–4.45 (m, 1H), 4.28 (dd, *J* = 11.5, 5.5 Hz, 1H), 4.23 (dd, *J* = 11.5, 7.3 Hz, 1H), 2.12 (s, 3H), 2.09 (s, 3H).

[(2R,3R,6S)-3-acetoxy-6-methoxy-5-nitro-3,6-dihydro-2H-pyran-2-yl] methyl acetate **(3p)**: colorless syrup. ^1^H NMR (500 MHz, CDCl_3_) δ 7.27 (d, *J* = 4.2 Hz, 1H), 5.55 (s, 1H), 5.39 (dd, *J* = 5.6, 2.8 Hz, 1H), 4.41–4.36 (m, 1H), 4.29–4.26 (m, 2H), 3.54 (s, 3H), 2.12 (s, 3H), 2.09 (s, 3H).

## Results and Discussion

### Condition Screening

We first applied different catalysts, including conventional Lewis acid, solid acid, and nanomagnetic catalysts, while the reaction of one equivalent of 3,4,6-tri-*O*-acetyl-2-deoxy-2-nitro-glucal (**1**) with 1.2 equivalents of EtOH (**2a**) was considered the model reaction. As summarized in Table 1, it was found that in DCM, the catalyst and temperature are the key factors through the screening of different catalysts at room temperature (entries 1–7) and 40°C (entries 8–14). Among them, the nanocatalyst Fe_3_O_4_@C@Fe(III) gave the best performance at 40°C in DCM with a yield of 72% (entry 10). Next, in order to improve the reaction efficacy, we examined various solvents commonly used in Ferrier reactions (DCE, DCM, MeCN, THF, and 1,4-dioxane) and extended the reaction temperature range to 60°C (entries 16–22). However, we found that the reaction effect was not as good as that of entry 10, despite the fact that the yields increased when the temperature increased in these solvents. Finally, we tried to adjust the catalyst amount (entries 23–26) and found the best catalyst equivalent that could afford the product with excellent yield (84%) was 0.3 (entry 24).

**TABLE 1:** Optimization of the Ferrier rearrangement reaction conditions.
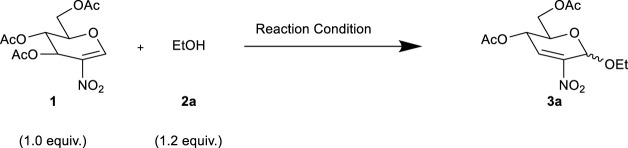

Entry	Catalyst^a^	Equivalent (catalyst)	Solvent	T (^o^C)	Time (h)	Yield (%)^b^
1	A	1.0	DCM	25	12	ND^c^
2	B	1.0	DCM	25	12	ND
3	C	1.0	DCM	25	12	ND
4	D	1.0	DCM	25	12	ND
5	E	1.0	DCM	25	12	<5
6	F	1.0	DCM	25	12	<5
7	G	1.0	DCM	25	12	<5
8	A	1.0	DCM	40	6	<5
9	B	1.0	DCM	40	6	<5
10	C	1.0	DCM	40	6	72
11	D	1.0	DCM	40	6	20
12	E	1.0	DCM	40	6	68
13	F	1.0	DCM	40	6	63
14	G	1.0	DCM	40	6	17
15	C	1.0	DCE	40	6	45
16	C	1.0	MeCN	40	6	<5
17	C	1.0	1,4-dioxane	40	6	ND
18	C	1.0	THF	40	6	28
19	C	1.0	DCE	60	4	68
20	C	1.0	MeCN	60	12	10
21	C	1.0	1,4-dioxane	60	12	ND
22	C	1.0	THF	60	6	50
23	C	0.5	DCM	40	6	75
24	C	0.3	DCM	40	6	84
25	C	0.1	DCM	40	8	60
26	C	0.3	DCM	25	12	15

a A: Fe_3_O_4_@C@SO_3_H; B: Fe_3_O_4_@C@Al (III); C: Fe_3_O_4_@C@Fe (III); D: FeCl_3_/C, E: FeCl_3_·6H_2_O/C; F: FeCl_3,_ G: FeCl_3_·6H_2_O.

b Isolated yield.

c Not detected.

### Substrate Extension

With the optimal conditions established, we investigated the reaction scope using a variety of acceptors. As summarized in Table 2, this magnetic core-shell catalyst could effectively promote the Ferrier rearrangement reaction with different alcohol acceptors (including simple alcohol, branched-chain alcohol, unsaturated alcohol, halogenated alcohol, complex natural alcohol, and sugar acceptors) by the good yield to obtain the corresponding 2-nitro-2,3-unsaturated glycoside. Compared with the literature studies, the yields of compounds **3b** (entry 2) and **3e** (entry 5) were increased by 20%. The yield of compound **3d** (entry 4) increased by 30% (Dharuman et al., 2013). Our previous research found that 2,3-unsaturated glycoside of hydroxymethylfurfural (HMF), which is a significant platform compound and valuable molecule from biomass materials, showed promising antitumor activities (Ding et al., 2018). Therefore, HMF was chosen as a special acceptor. The yield could reach 79% in this system, as shown in entry 7. Not only that, the glycosyl acceptor (entry 9) could be well-applied to the reaction system, and the yield could reach 83%. Moreover, isopropanol gave a fairly good yield in our system compared with that in the reported methods (Jiang et al., 2021). From entry 10 and entry 11, for complex natural alcohols, such as menthol and cholesterol, the reaction time was prolonged, and the yield was obtained with 71–73%. Phenolic acceptors, a type of difficult acceptors in the previous Ferrier rearrangement research, such as phenol and *p*-methoxyphenol, could also produce the corresponding phenolic glycosides with excellent yield at 0.1 equivalent (Dharuman et al., 2013; Jiang et al., 2021). Compared with the literature studies, the yield of the corresponding product **3m** (entry 13) was also increased by 10% (Dharuman et al., 2013). In addition, it could be found from entries 12–14 that the reaction rate of *p*-methoxyphenol was higher than that of the phenol acceptor, while the reaction rate of *p*-bromophenol (entry 14) was almost zero, indicating that the electronic effect of the substituent groups on the phenolic acceptor has a significant influence on the reaction results. In addition, the reaction scope was tested with galactose donors **1b,** gaining **3o–3p** with good yields.

**TABLE 2:** Substrate extension of the Ferrier rearrangement reaction based on 2-nitroglucal donors.
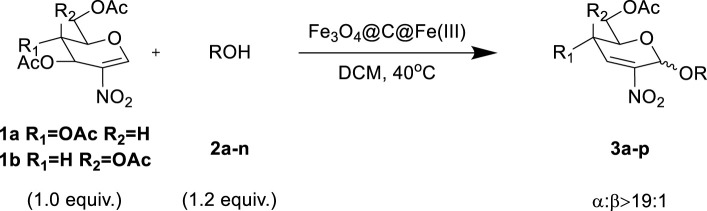

Entry	Acceptors	Products	Time (h)	Yield (%)^a^
1	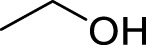	**3a**	6	84
2	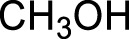	**3b**	6	82
3	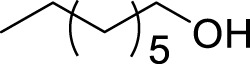	**3c**	6	81
4	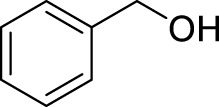	**3d**	6	89
5	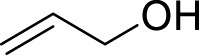	**3e**	6	84
6	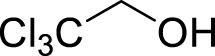	**3f**	6	81
7	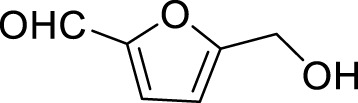	**3g**	6	79
8	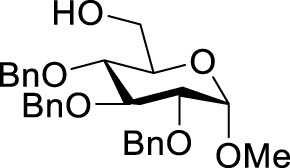	**3h**	6	83
9	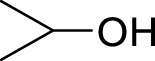	**3i**	6	86
10	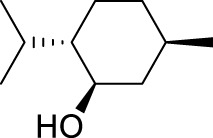	**3j**	8	73
11	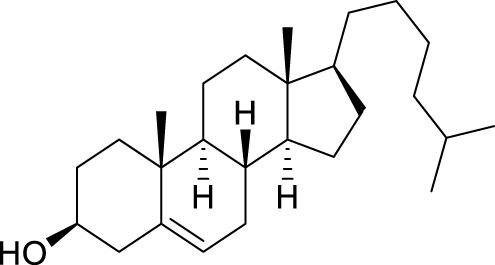	**3k**	24	71
12	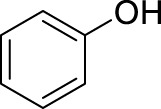	**3l**	8	80
13	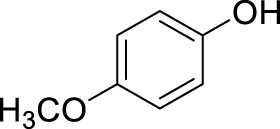	**3m**	6	86
14	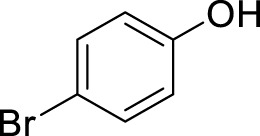	**3n**	24	Trace
15^b^	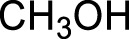	**3o**	12	84
16^b^	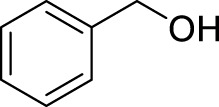	**3p**	12	79

a Isolated yield.

b Donor is galactosyl **1b**.

The structure and stereochemistry of the rearrangement products were elucidated by NMR and mass spectroscopy and were subsequently compared with the reported data (Dharuman et al., 2013; Lafuente et al., 2019; Jiang et al., 2021). It was noteworthy that the stereoselectivity of 2-nitro-2,3-unsaturated glycoside compounds was confirmed as α: *β* > 19:1 by ^1^H NMR.

### Recycle Experiment

**FIGURE 2:**
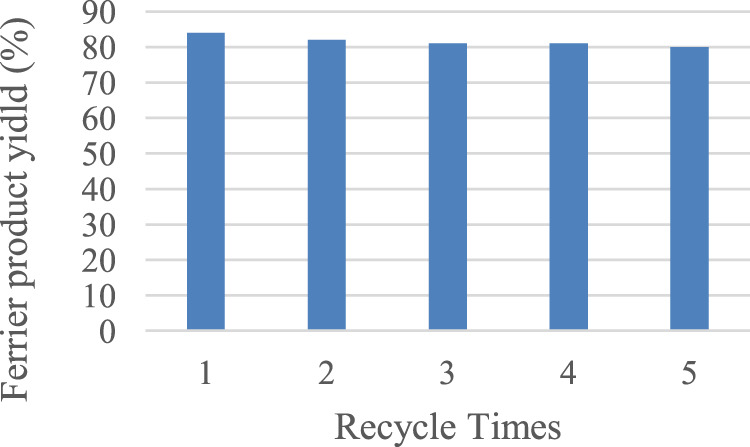
Recycling experiments of Fe_3_O_4_@C@Fe(III). Reaction conditions: 1 (0.05 mmol, 15.85 mg), EtOH (0.06 mmol, 3.6 µL), Fe_3_O_4_@C@Fe(III) (34 mg), and DCM (0.5 ml), 6 h, 40°C.

As a catalyst with superparamagenetic nano-sized Fe_3_O_4_ nuclei, our catalyst is supposed to be recycled efficiently with an external magnet. We tested the recycling performance of the catalyst by using the model reaction to examine whether the ferric ion remained tightly bound to the core-shell Fe_3_O_4_@C under heated conditions. As shown in Figure 2, the product was still obtained in 80% yield even after the catalyst had been recycled five times. The catalyst Fe_3_O_4_@C@Fe(III) is suitable for the reaction system and has good recycle performance.

### Mechanism Discussion

**FIGURE 3:**
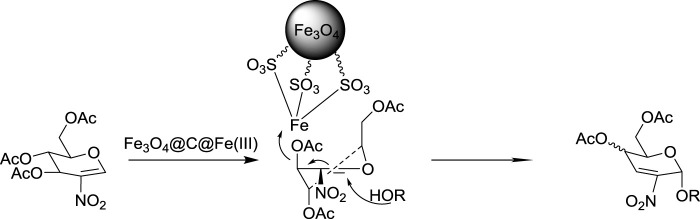
Plausible mechanism.

Based on the experimental results and literature studies, a reaction mechanism may be proposed as follows (Figure 3). (Dharuman et al., 2013; Lafuente et al., 2019; Jiang et al., 2020b; Jiang et al., 2021): As we know, a halogen substituent at the C-2 position of the glycals has a remarkable effect, and those electron-withdrawing groups would reduce the electron density of the oxocarbenium intermediate, which is commonly assumed as the intermediate cation in the Ferrier rearrangement (Dong et al., 2019). Thus, under the nanocatalysis of Fe_3_O_4_@C@Fe(III), C3 site deacylation in 2-nitro-glucal would occur very slowly and electron transfer would occur in a different way, with the aid of the nucleophiles. At the same time, due to the high steric hindrance of nanocatalysts, the nucleophilic receptor would have to attack the activated 2-nitroglycal from the α side to obtain the α-product *via* S_N_2’ mechanism (Lalic et al., 2008). Differing from the usual mechanism (Lalic et al., 2008), there is much fewer oxonium intermediates generated in our system, so the Ferrier-rearranged product (Lalic et al., 2008) was obtained with much higher stereoselectivity than that of the normal glycals and halogenated glycals.

## Conclusion

In summary, we have successfully applied a nano-sized magnetic catalyst Fe_3_O_4_@C@Fe(III) for Ferrier rearrangement of 2-nitro glucal. This core-shell catalyst successfully solved the problems of catalyst separation, circulation, and substrate suitability. In the reaction system, substrates are widely applicable to various alcohols especially long-chain alcohols, branched-chain alcohols, unsaturated alcohols, halogenated alcohols, cyclic alcohols, steroidal alcohols, and carbohydrate acceptors. In addition, phenols could be applied in good yields. Furthermore, all the products could be achieved with a high stereoselectivity of α: *β* > 19:1. These results demonstrated that the catalyst and the optimal reaction condition are broadly applicable to different Ferrier rearrangement acceptors and have, thus, built the foundation for the synthesis of natural bioactive molecules and their analogs.

## Data Availability

The original contributions presented in the study are included in the article/Supplementary Material, further inquiries can be directed to the corresponding author.
