# Supplementary material for: Synthesis of 2-Nitro-2,3-Unsaturated Glycosides by a Nanomagnetic Catalyst Fe3O4@C@Fe(III)
Source: Front Chem. 2022 May 11;10:865012. doi: 10.3389/fchem.2022.865012 (PMC9130751; doi:10.3389/fchem.2022.865012)

Supporting Information

**Synthesis of** **2-nitro-2,3-unsaturated glycosides by a magnetic catalyst** **Fe_3_O_4_@C@Fe(III)**

Yu Yang, ^[1]^ Nan Jiang, ^[1]^ Yuling Mei, ^[1]^ Zekun Ding, ^[1]^ and Jianbo Zhang * ^[1]^

School of Chemistry and Molecular Engineering, East China Normal University, Shanghai, 200241, China

E-mail: [jbzhang@chem.ecnu.edu.cn](mailto:jbzhang@chem.ecnu.edu.cn)

**Contents** Page Number

General information………………………………………………………………….…………….2

Catalyst preparation………………………………………………………………………….…….2

Synthesis of 2-nitroglycal…………………………………………………………………….……2

General procedure for the synthesis of 2-nitro-2,3-unsaturated glycosides……………….…….....3

Copies of NMR Spectra (1a, 3a-3m) …………………………………………………..…….....4-17

Copies of HRMS Spectra (3a-3m) …………………………..………………………………...18-24

**General information**

All reactions were carried out under dry nitrogen atmosphere. All solvents and reagents were obtained from commercial sources unless otherwise stated and were purified according to standard procedures. Removal of solvent in vacuo refers to distillation using a rotary evaporator attached toan efficient vacuum pump. ^1^H NMR and ^13^C NMR spectra were recorded on Bruker DRX-500 NMR spectrometer in solutions of CDCl_3_ using tetramethylsilane as the internal standard. Mass spectra were determined on LTQ-XL (Thermo scientific, USA) with an (ESI) Ion trap mass spectrometer.

**Catalyst preparation**

Lotus leaves were collected from Yingtao river in the East China Normal University, Minghang Campus and washed five times repeatedly with distilled water to remove any surface contamination. The washed leaves were dried for 24h at 60°C in vacuum drying oven and then ground into a fine powder. Next, 5g powder was added to 125 mL deionized water and soaked for 24h. The soaked mixture was heated to 80 °C for 6h and then reduced pressure distilled to remove water.10 The residue was fully dried at 60°C in vacuum drying oven and we obtained 1.2g extract fully dissolved in 2mL PEG400 by ultrasonic for ca. 15min. Then the commercial available nanoFe_3_O_4_ (99.5%, 20nm beads, Aladdin Co. Ltd., China) and H_2_SO_4_ (2.0 mL, 98 wt.-%) were successively added with careful stirring. After ultrasonication for ca. 15 min, the products were washed with deionized water (six times) and acetone (twice) and dried under vacuum at room temperature for 6h. Finally, the obtained modified core-shell Fe_3_O_4_@C microparticles were immersed into CH_2_Cl_2_ solution of FeCl_3_ (0.5 mg/mL) for 12h and the final products were washed with CH_2_Cl_2_ (three times). The Fe_3_O_4_@C@Fe(III) catalyst was further dried and then stored in desiccators. The content of Fe^3+^ in the catalyst was calculated by complexometric titration method.

**Procedure for the synthesis of 2-nitroglycal**

General procedure for the synthesis of 2-nitroglycal: To a stirred solution of a glycal (1 mmol) and AgNO_3_ (1 mmol) in acetonitrile (10.0 mL) at 0°C was added dropwise acetyl chloride (1 mmol). After the completion of the addition, the reaction was stirred at 55°C monitored by TLC (PE/EA, 2:1) until the glycal donor was consumed completely. The reaction mixture was brought to room temperature and was neutralized to pH 7 by the addition of solid NaHCO_3_. The suspension was filtered and concentrated. The solvent was removed under reduced pressure to afford a crude product which was purified by silica gel flash chromatography with a gradient solvent system (PE/EA, 4:1) to yield 2-nitroglycals.

**General procedure for the synthesis of 2-nitro-2,3-unsaturated glycosides**

General procedure for the synthesis of 2-nitro-2,3-unsaturated glycosides: To a mixture of 2-nitroglycal (63.4 mg, 0.2 mmol), acceptor (0.24 mmol), nano magnetic catalyst Fe3O4@C@Fe(III) (0.06 mmol) were added DCM (2.0 mL) under an atmosphere of nitrogen. The reaction mixture was stirred at room temperature and monitored by TLC (PE/EA, 2:1) until the glycal donor was consumed completely. The solvent was removed under reduced pressure to afford a crude product which was purified by silica gel flash chromatography with a gradient solvent system (PE/EA, 4:1) to yield 2-nitro-2, 3-unsaturated glycosides.

^1^H NMR (500 MHz, Chloroform-d) **1a**


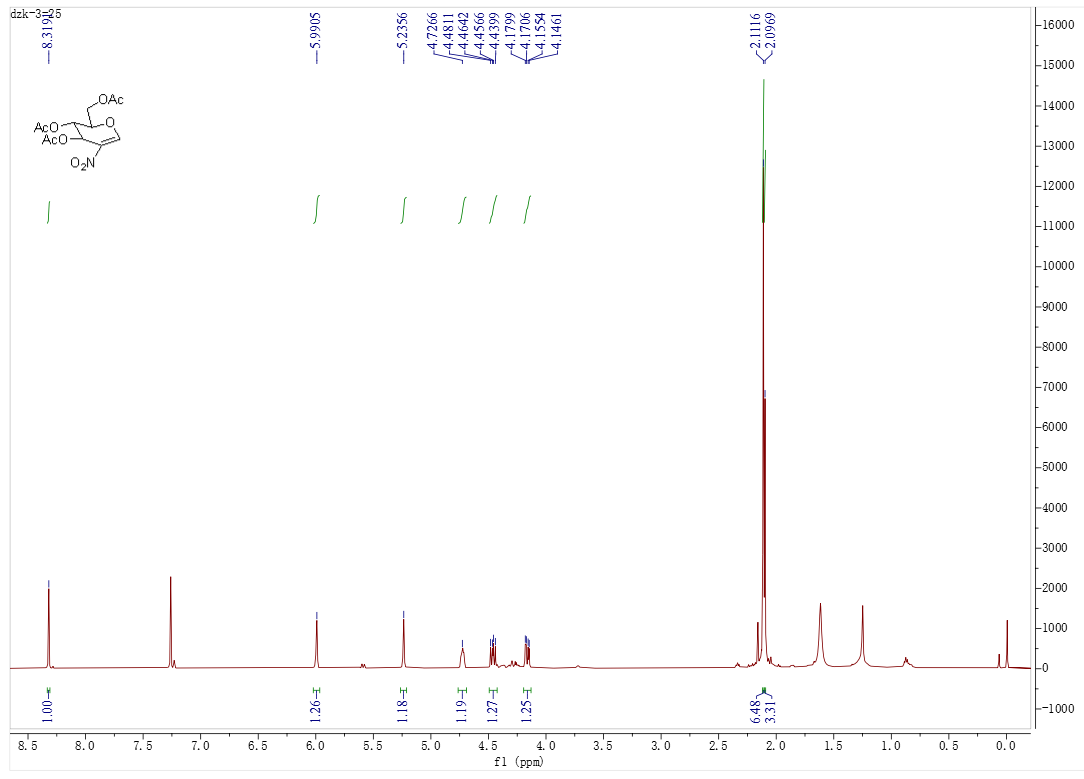


^1^H NMR (500 MHz, Chloroform-d) **3a**


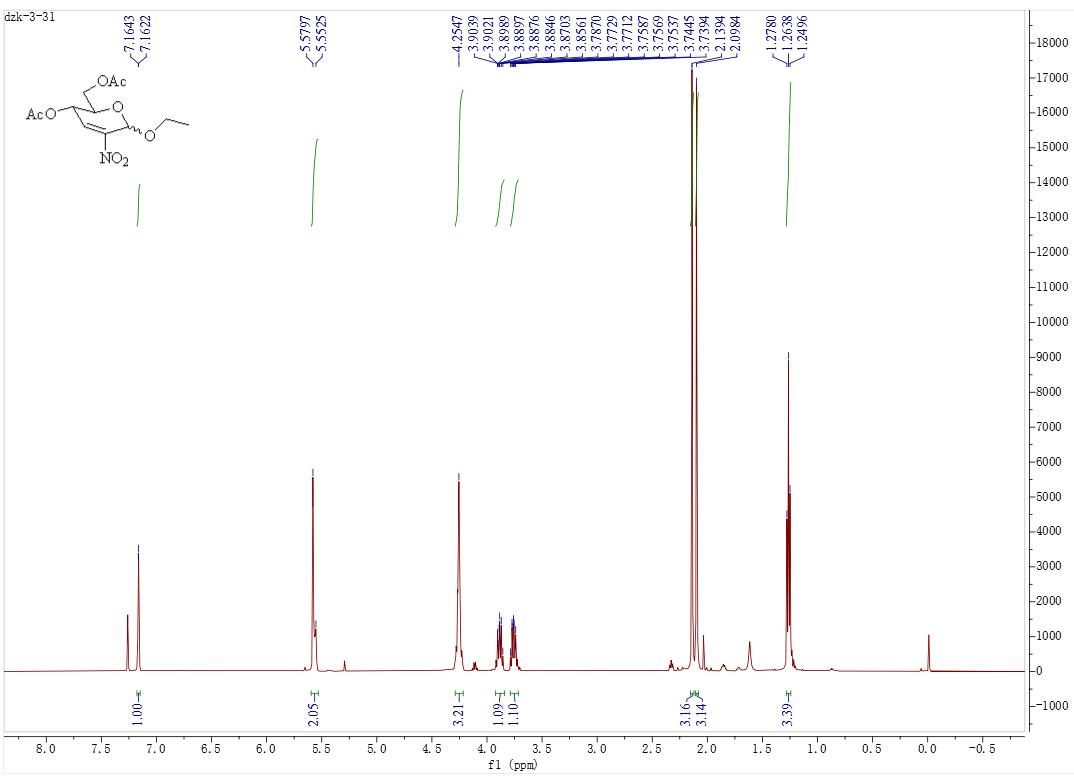


^13^C NMR (126 MHz, Chloroform-d)


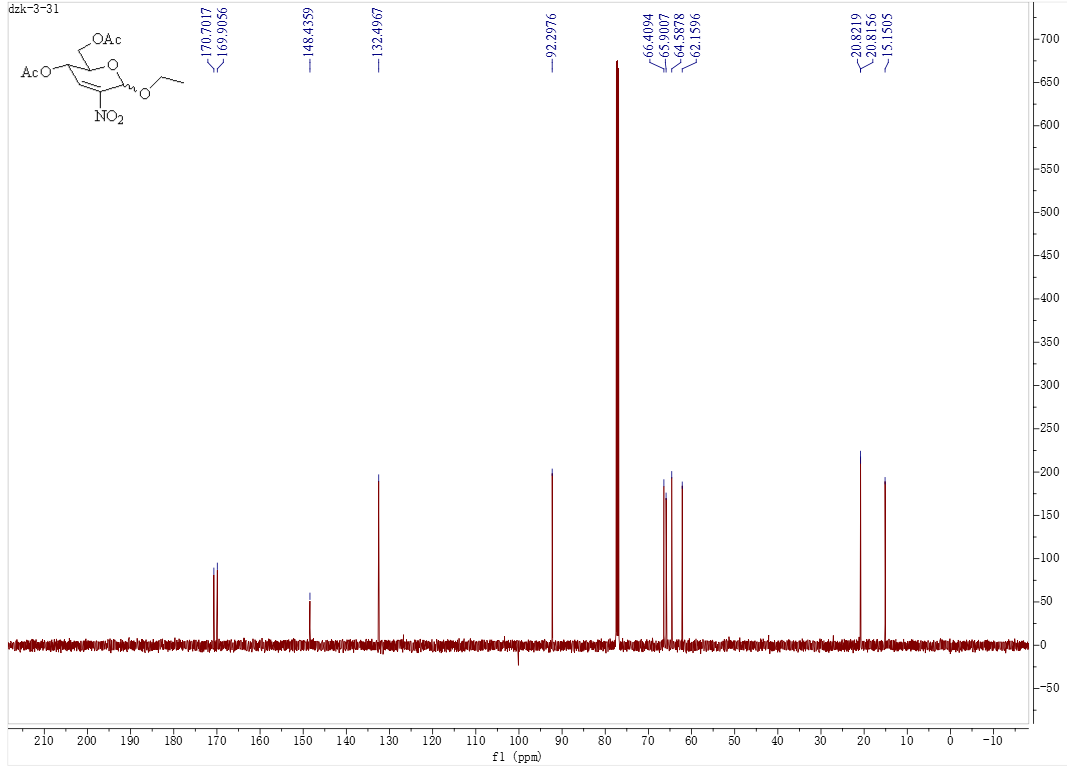


^1^H NMR (500 MHz, Chloroform-d) **3b**


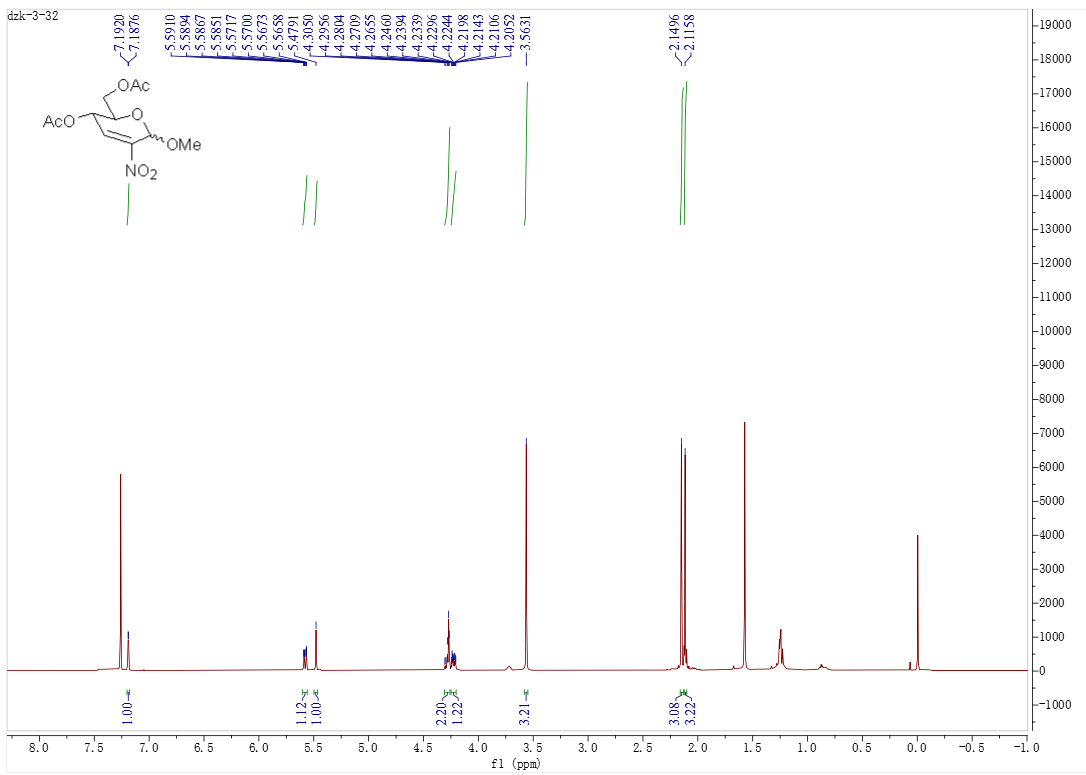


^13^C NMR (126 MHz, Chloroform-d)


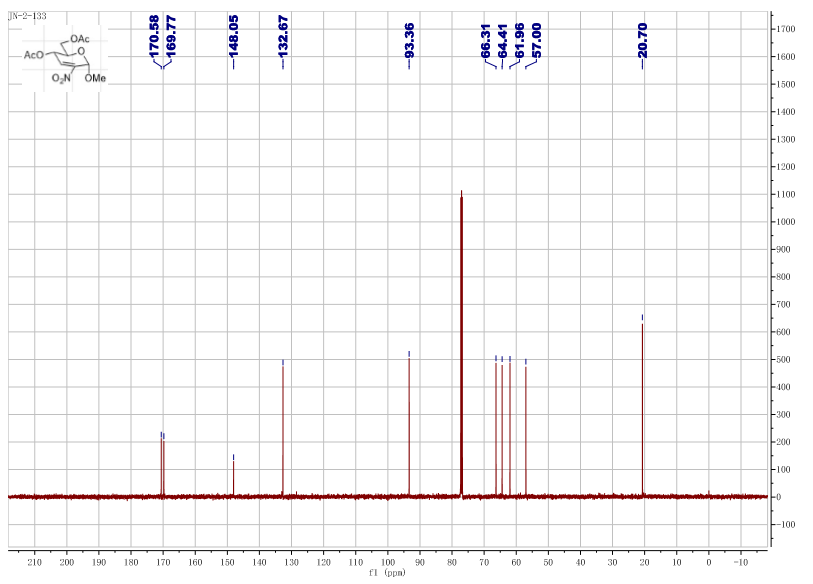


^1^H NMR (500 MHz, Chloroform-d) **3c**


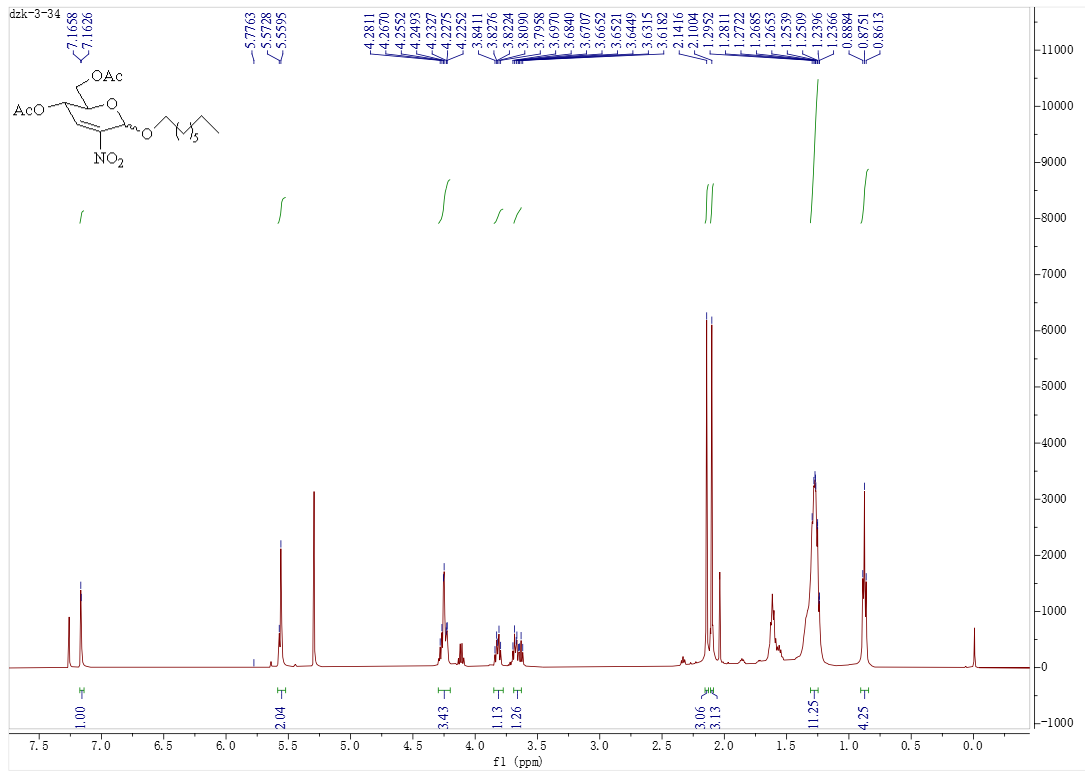


^13^C NMR (126 MHz, Chloroform-d)


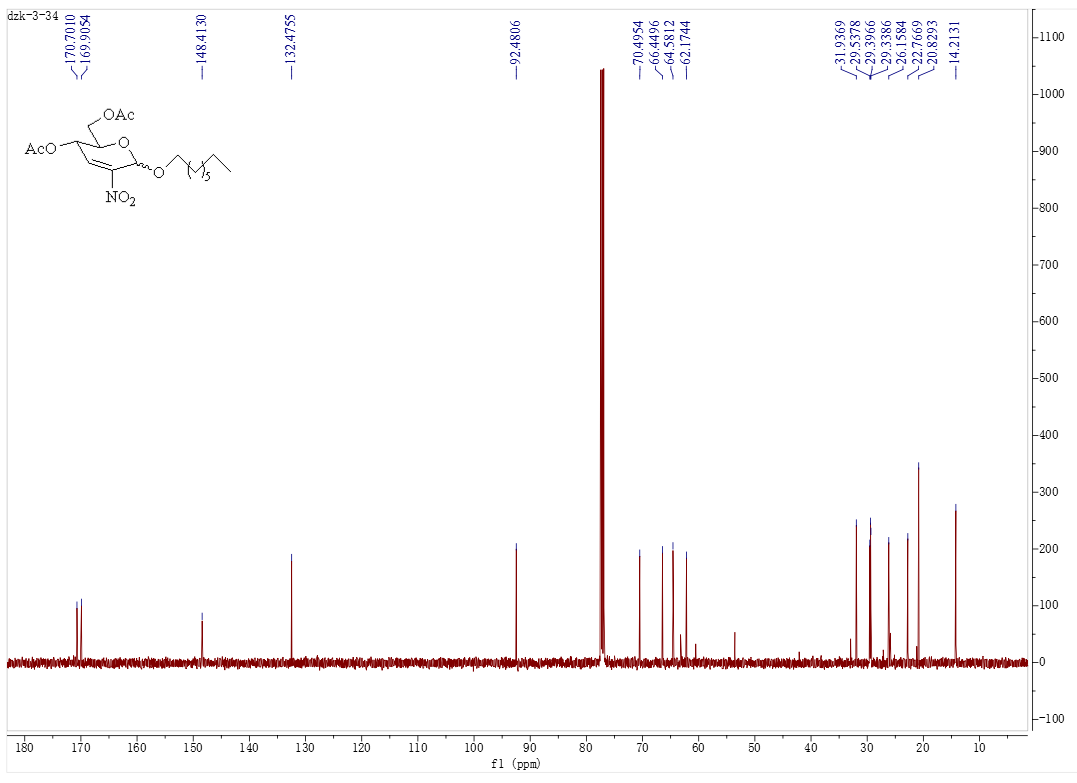


^1^H NMR (500 MHz, Chloroform-d) **3d**


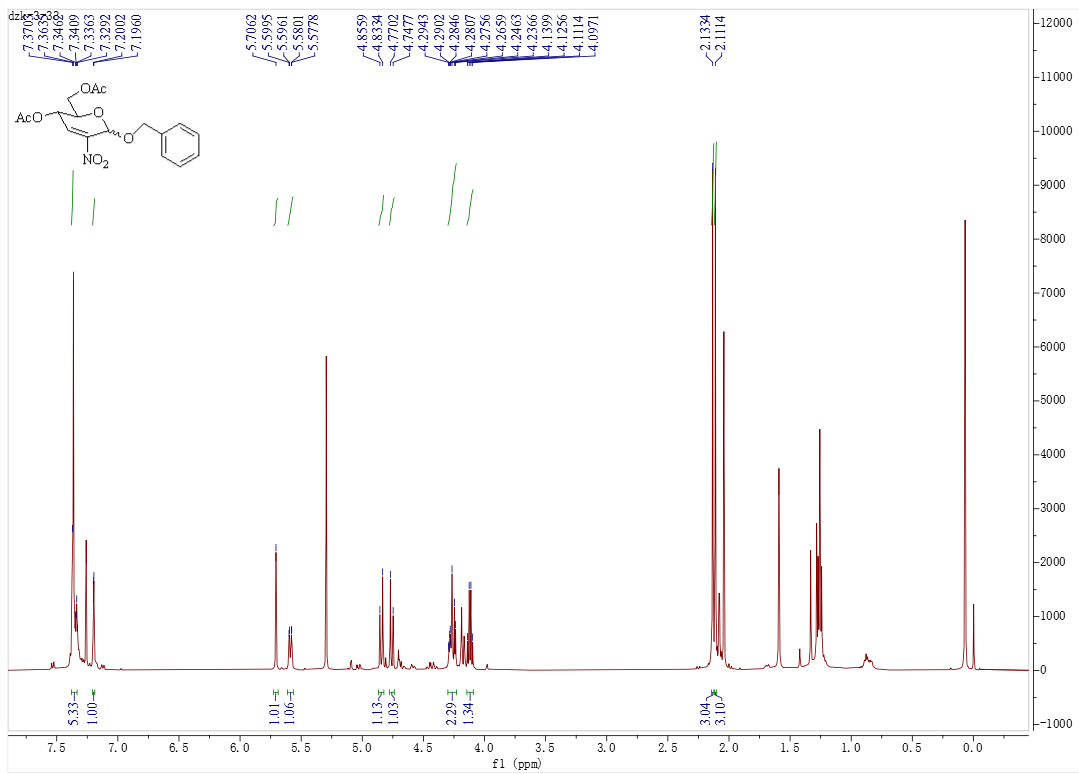


^13^C NMR (126 MHz, Chloroform-d)


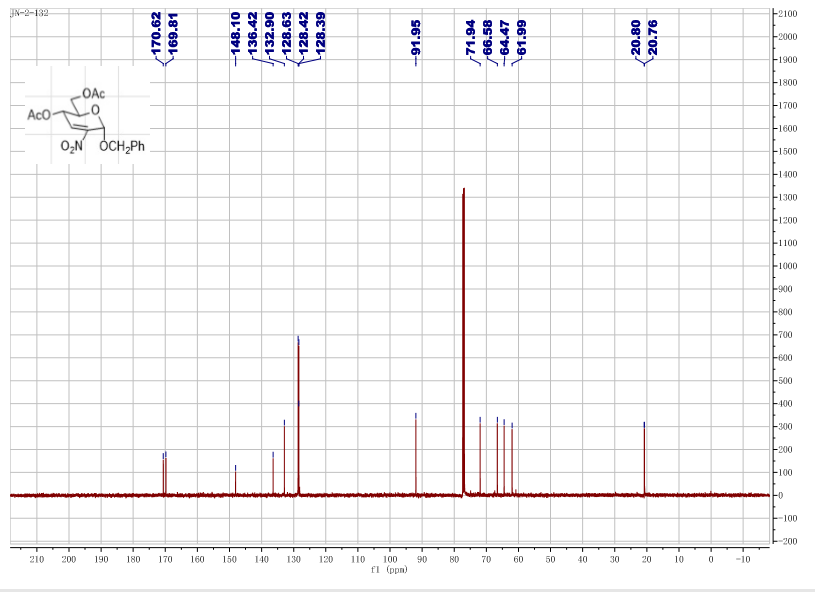


^1^H NMR (500 MHz, Chloroform-d) **3e**


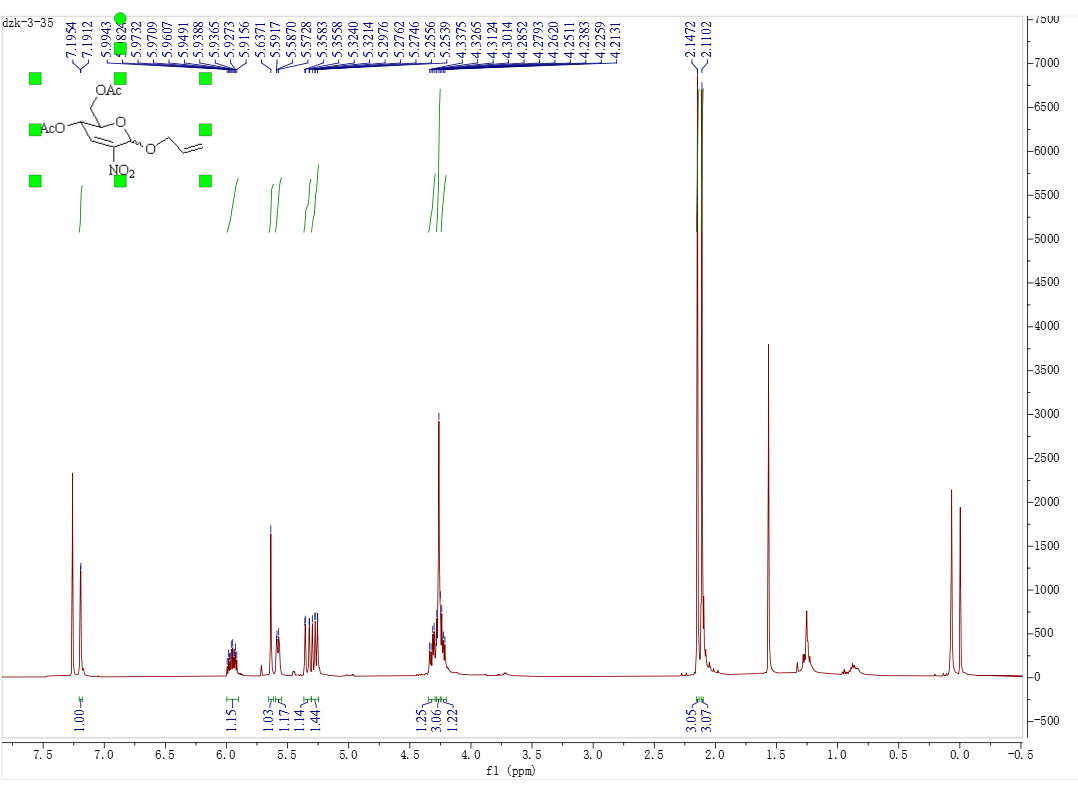


^1^H NMR (500 MHz, Chloroform-d) **3f**


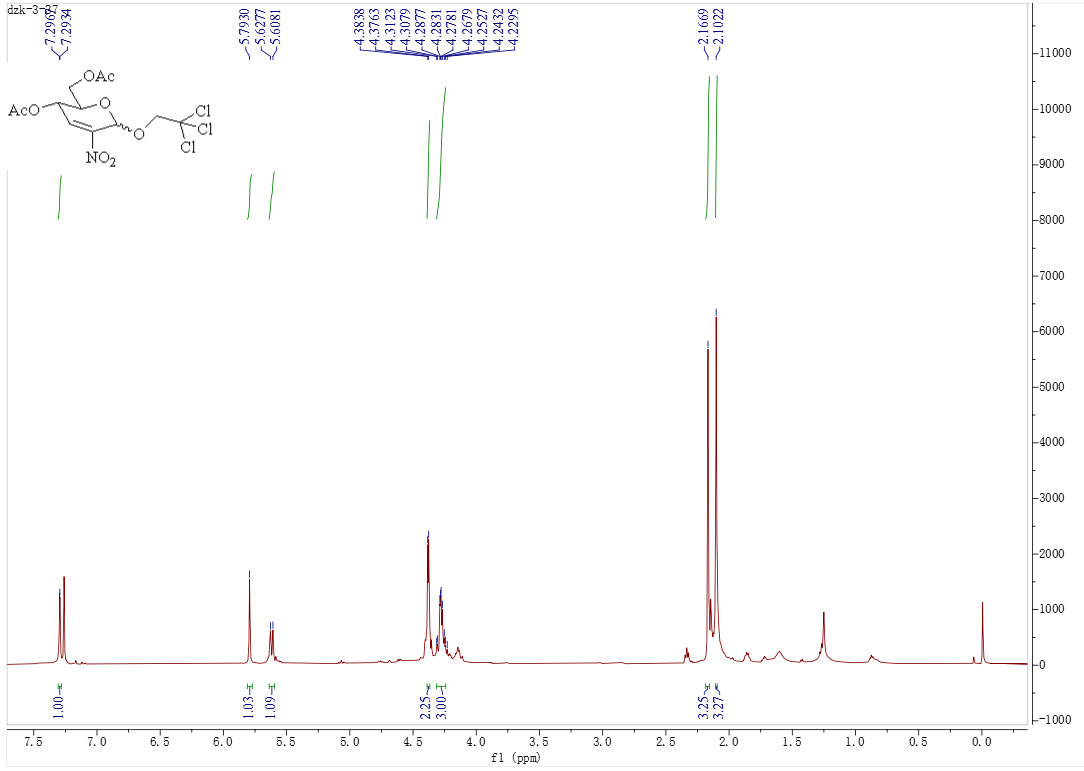


^13^C NMR (126 MHz, Chloroform-d)


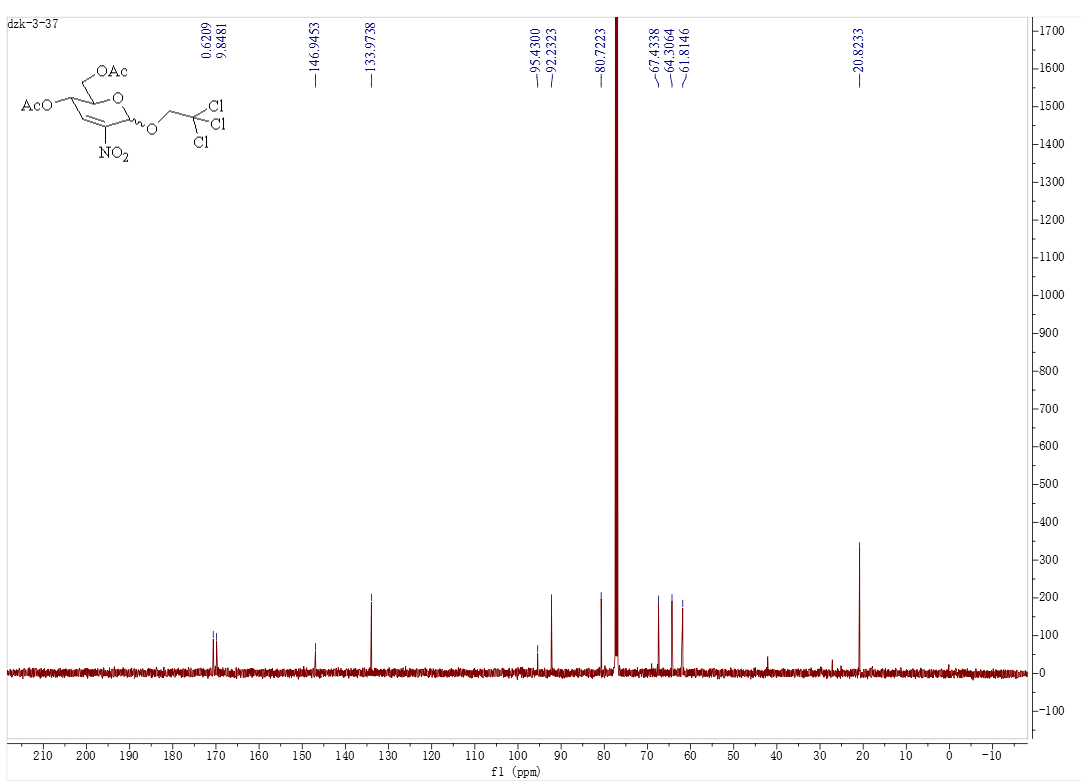


^1^H NMR (500 MHz, Chloroform-d) **3g**


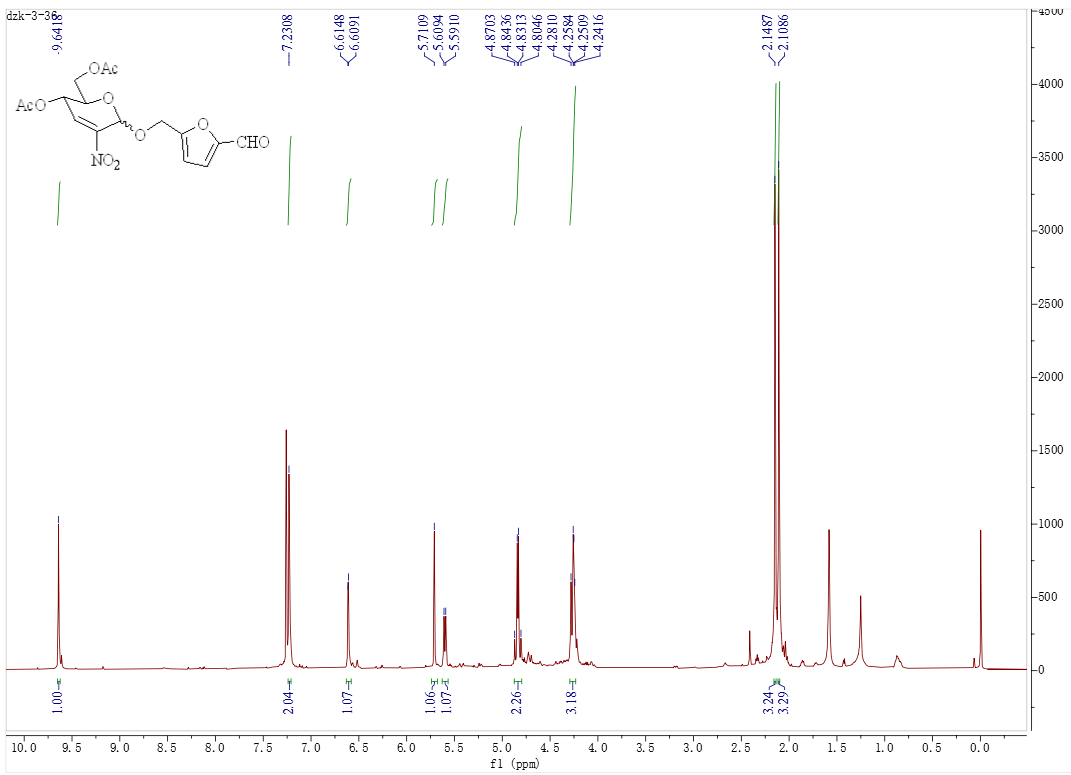


^13^C NMR (126 MHz, Chloroform-d)


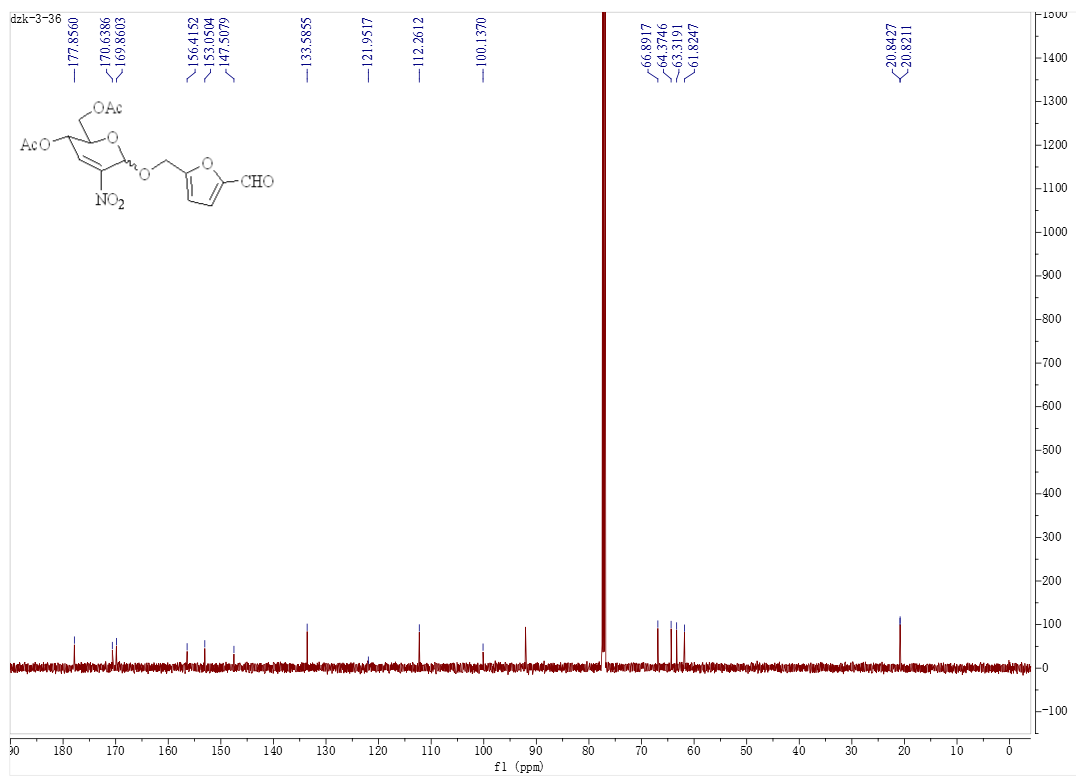


^1^H NMR (500 MHz, Chloroform-d) **3h**


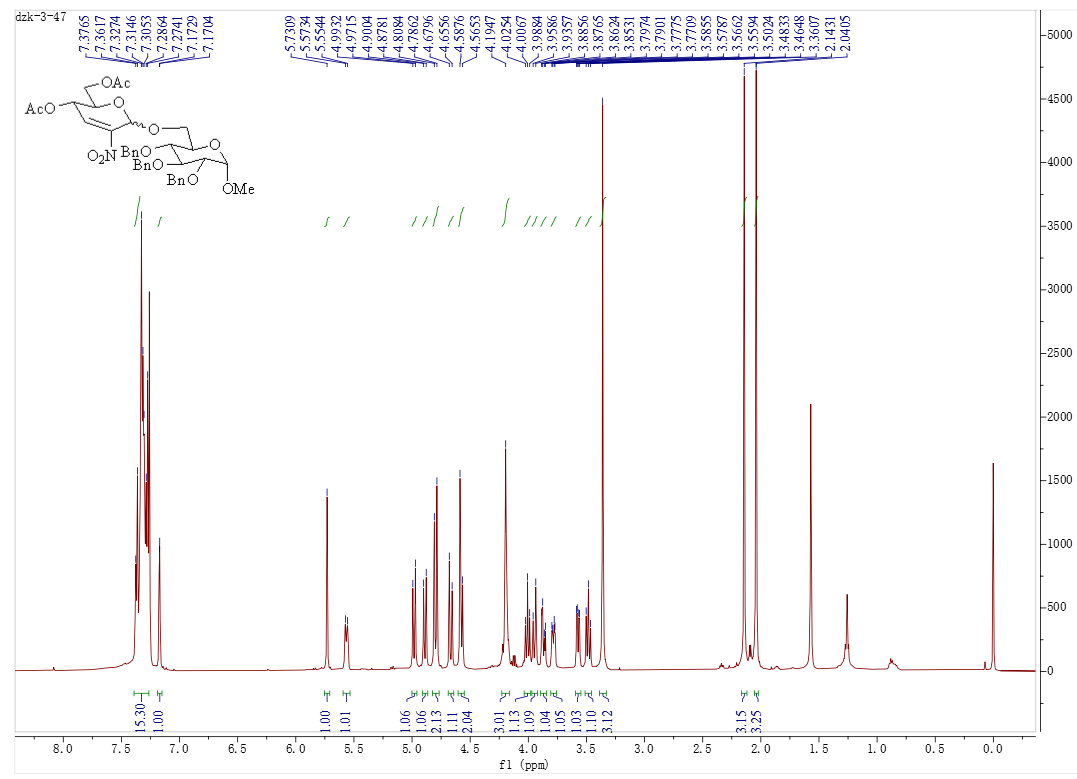


^13^C NMR (126 MHz, Chloroform-d)


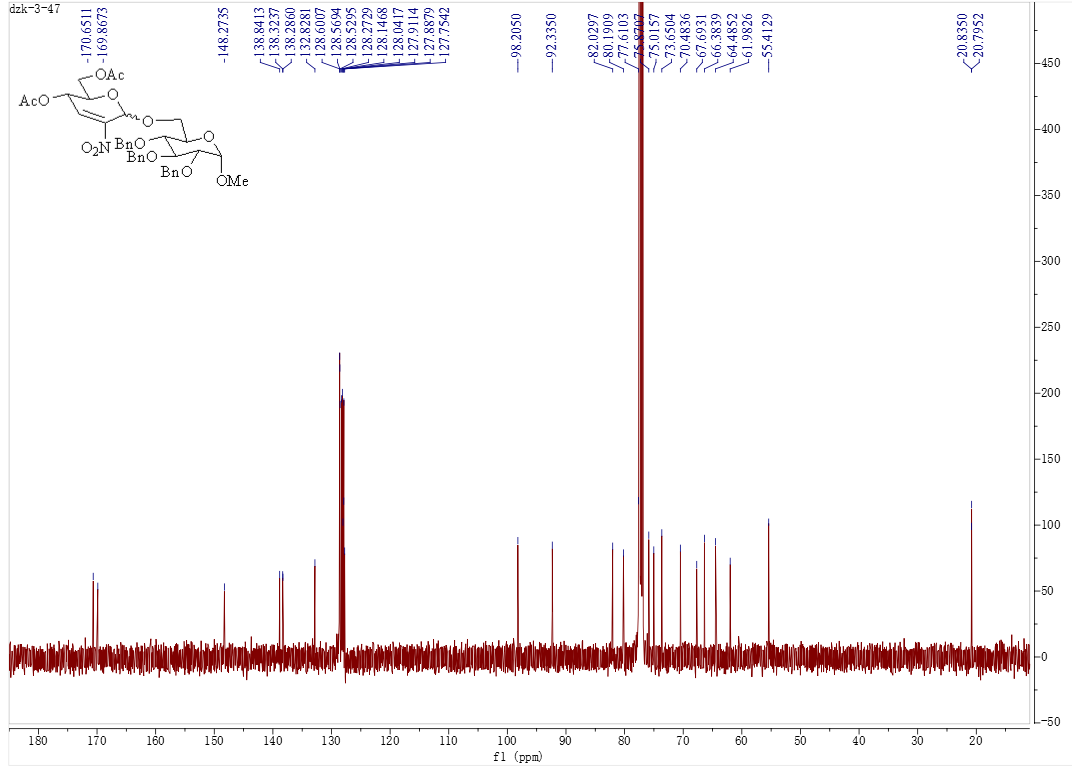


^1^H NMR (500 MHz, Chloroform-d) **3i**


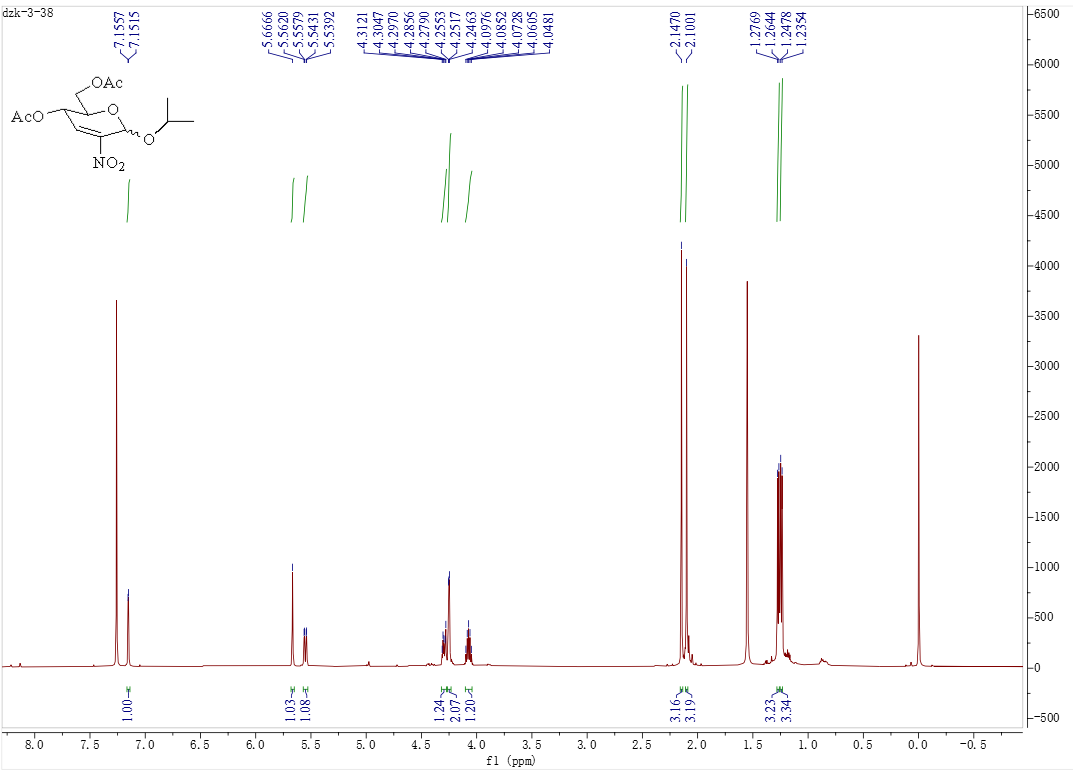


^13^C NMR (126 MHz, Chloroform-d)


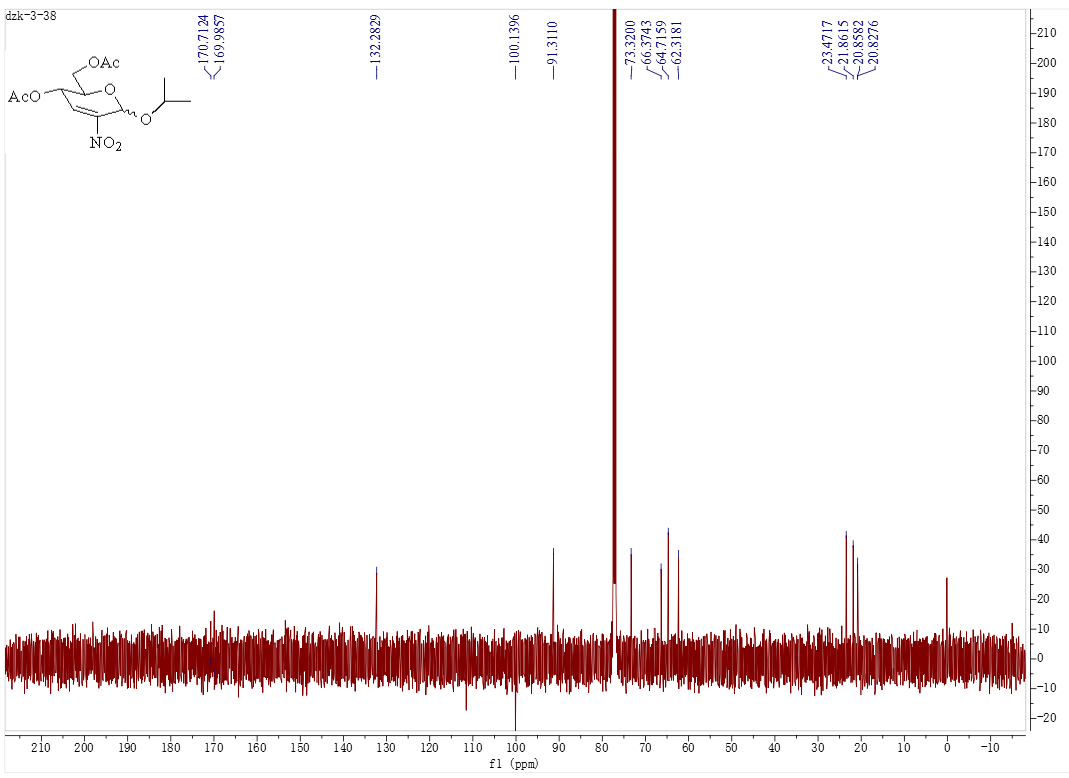


^1^H NMR (500 MHz, Chloroform-d) **3j**


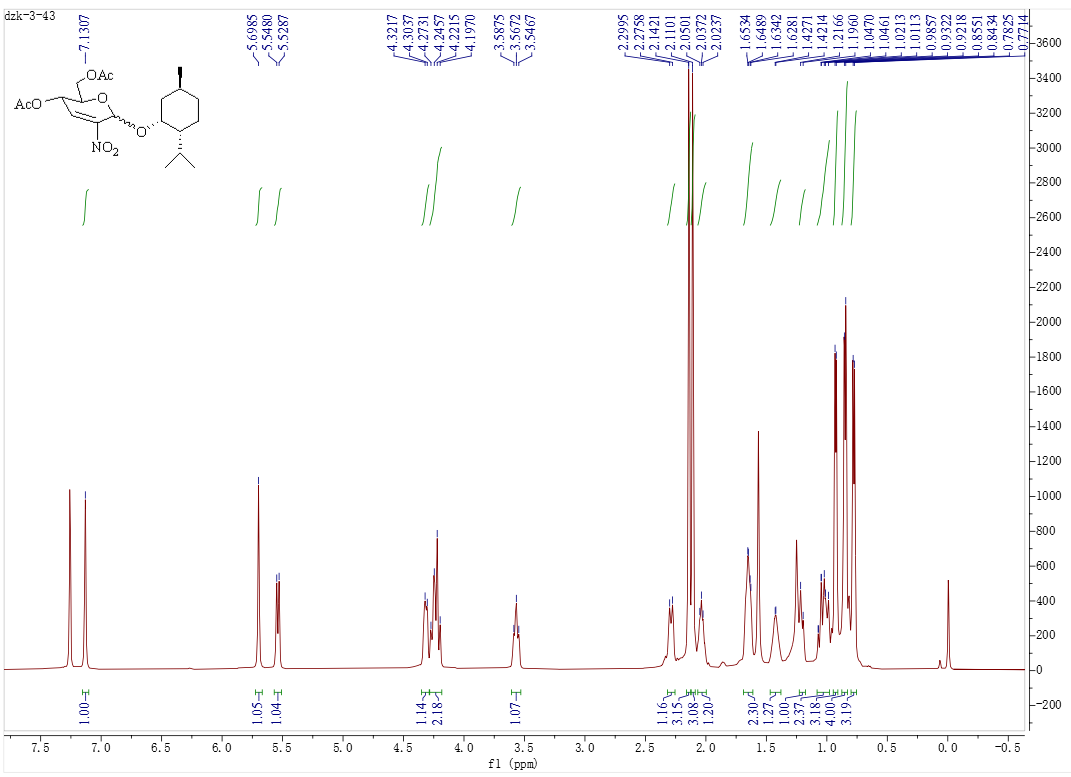


^13^C NMR (126 MHz, Chloroform-d)


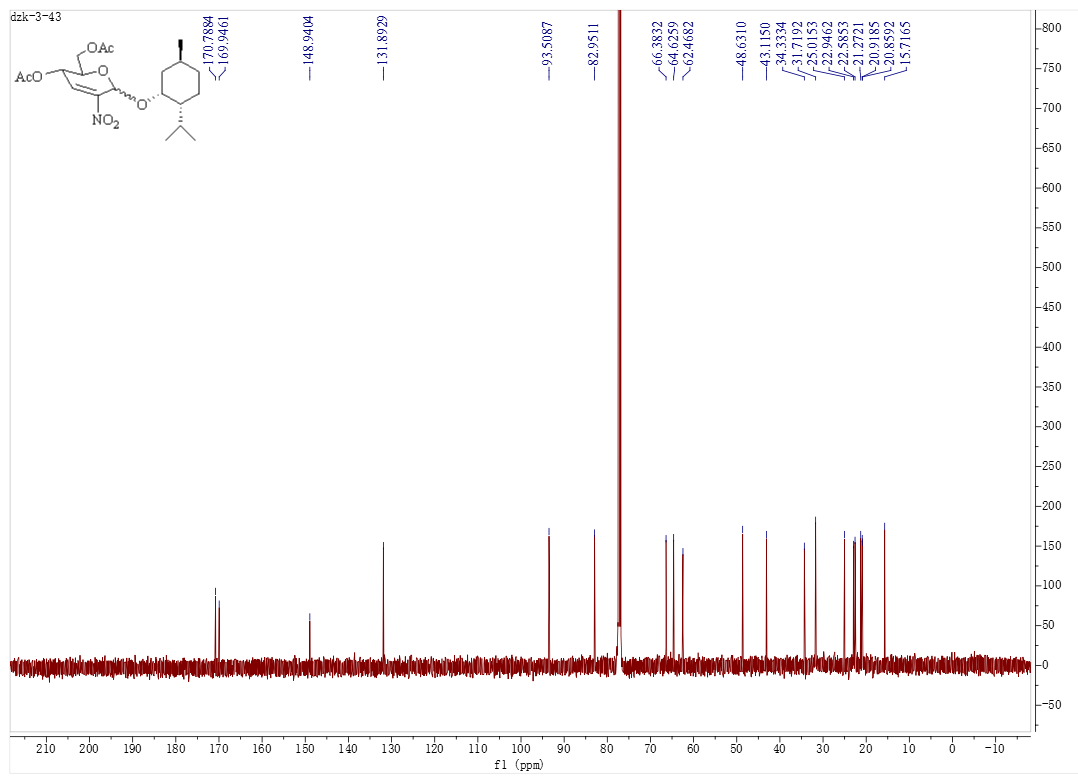


^1^H NMR (500 MHz, Chloroform-d) **3k**


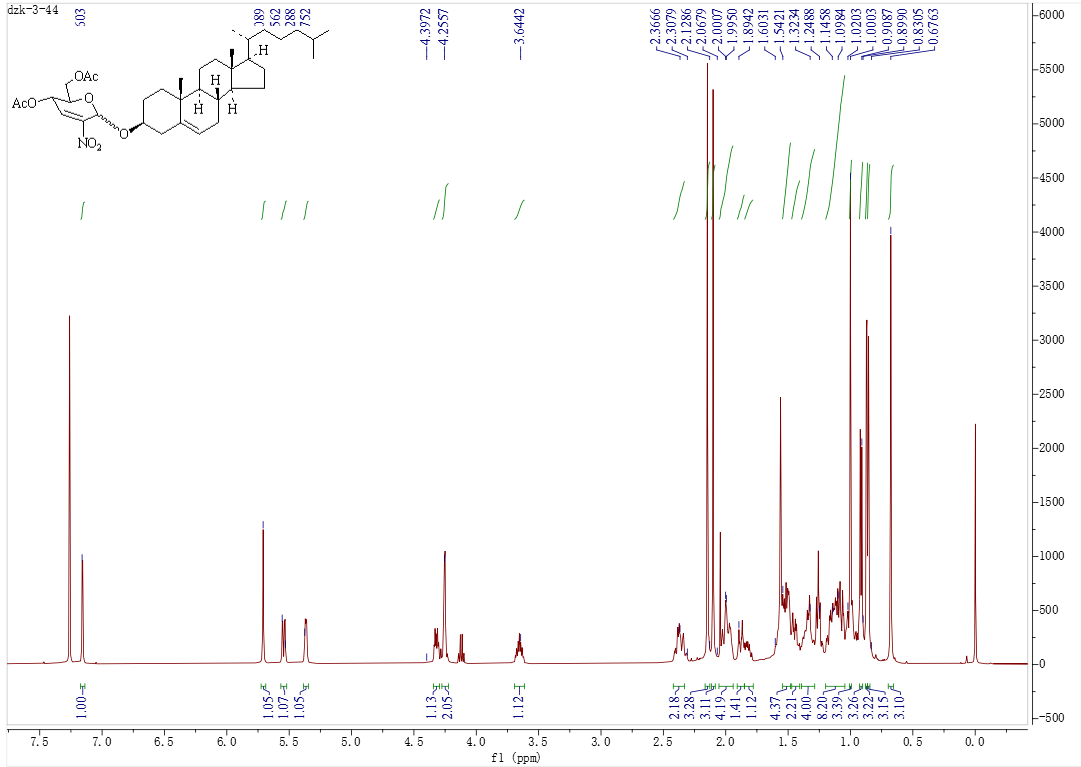


^13^C NMR (126 MHz, Chloroform-d)


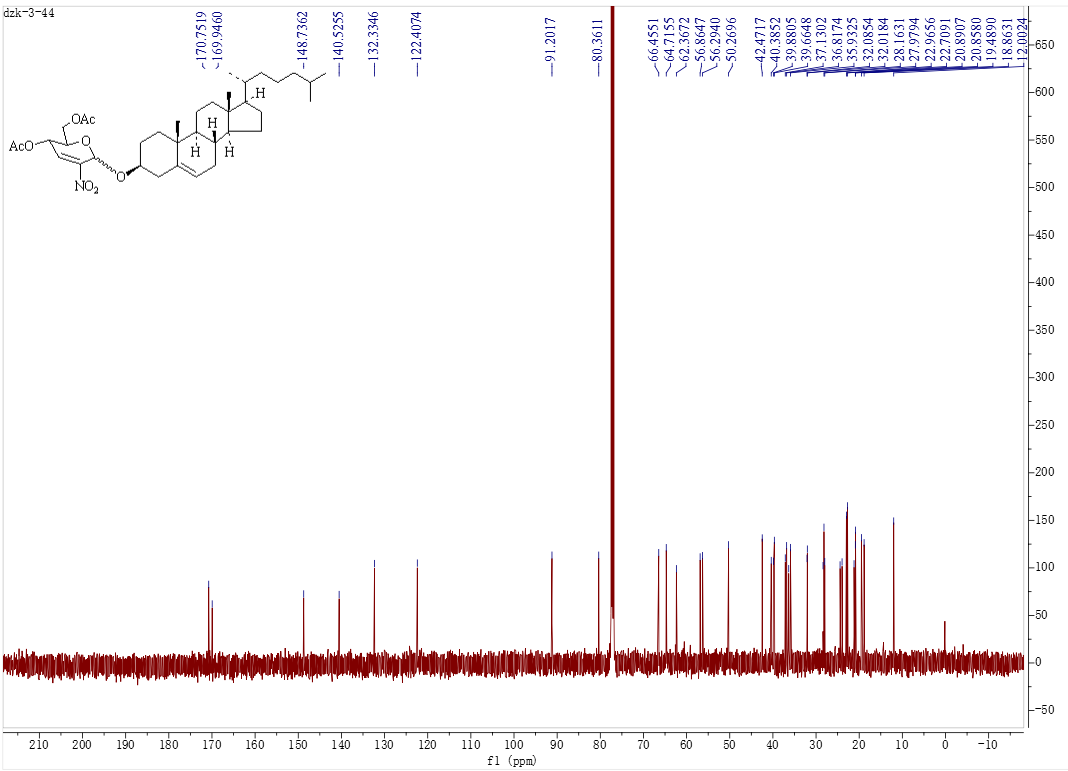


^1^H NMR (500 MHz, Chloroform-d) **3l**

**
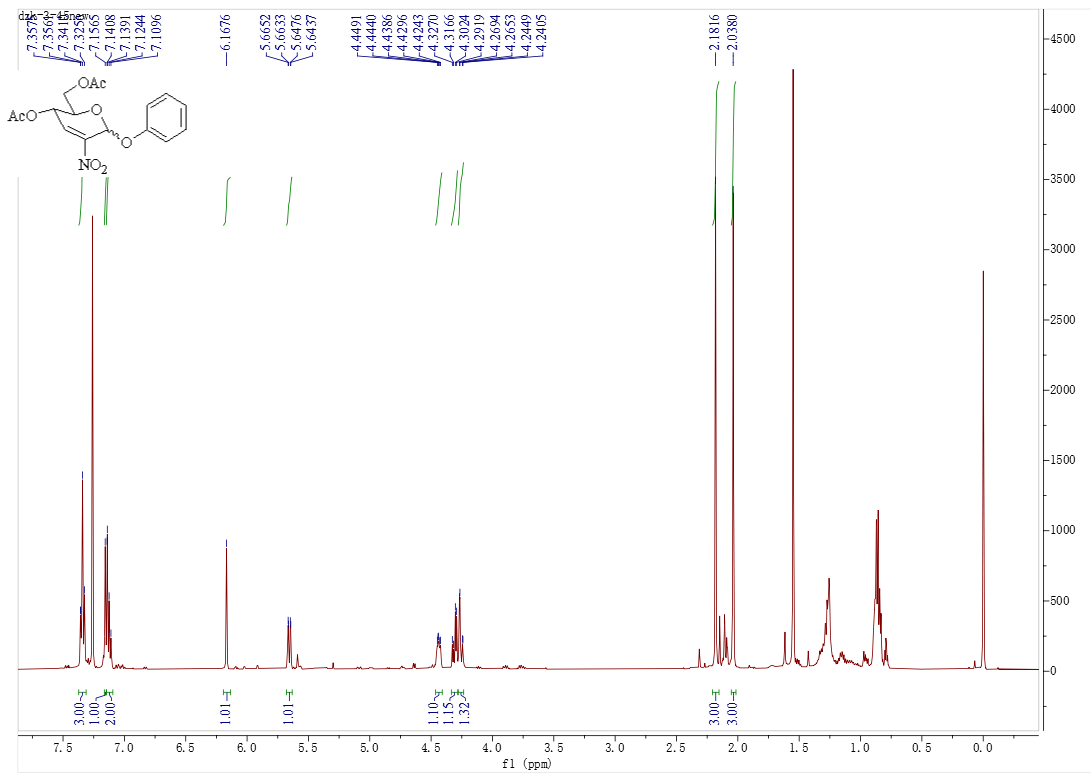
**

^13^C NMR (126 MHz, Chloroform-d)


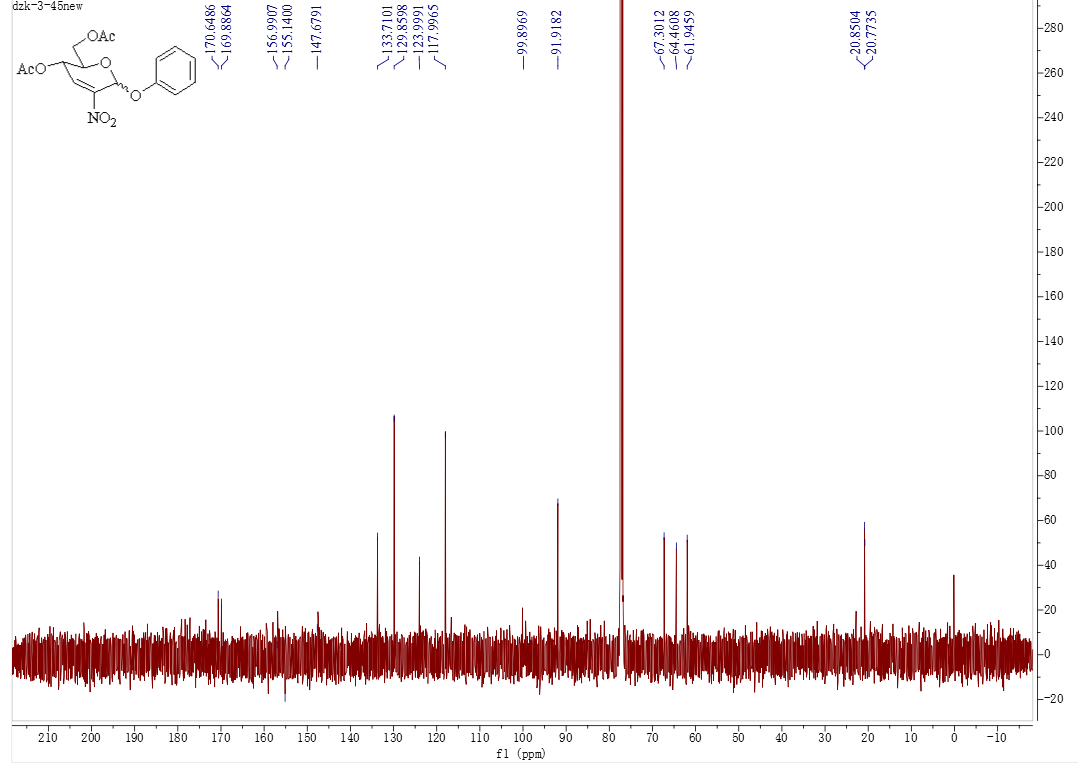


^1^H NMR (500 MHz, Chloroform-d) **3m**

**
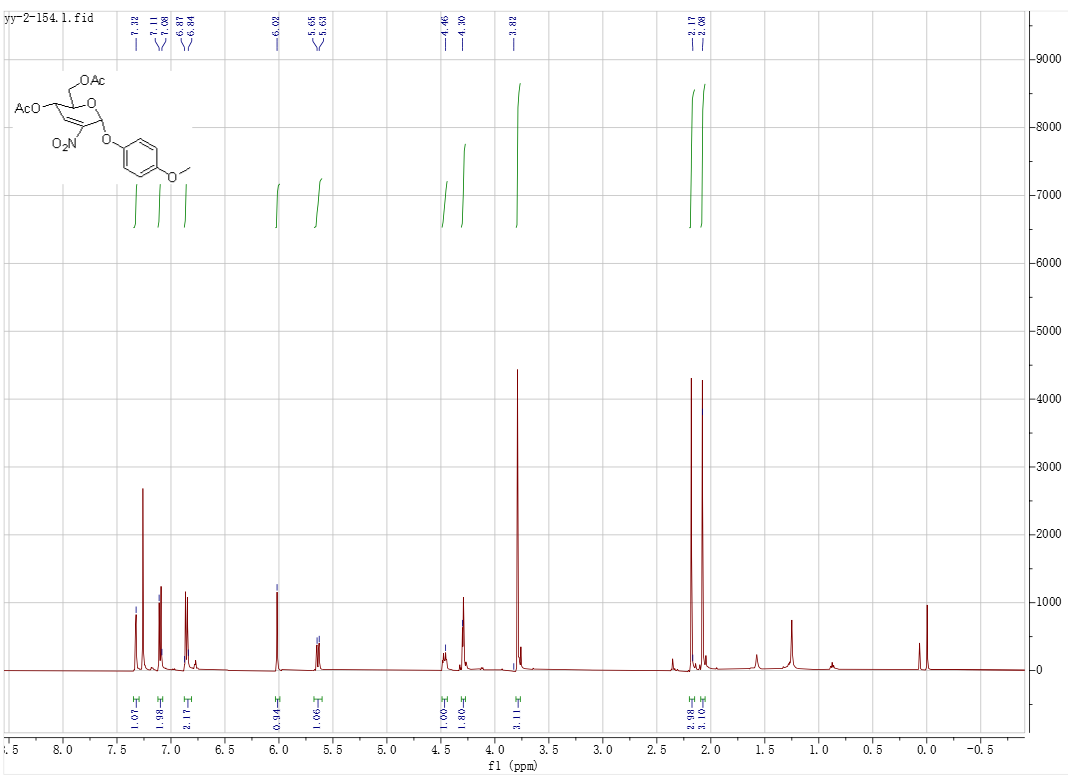
**

HRMS data

**3a**

**
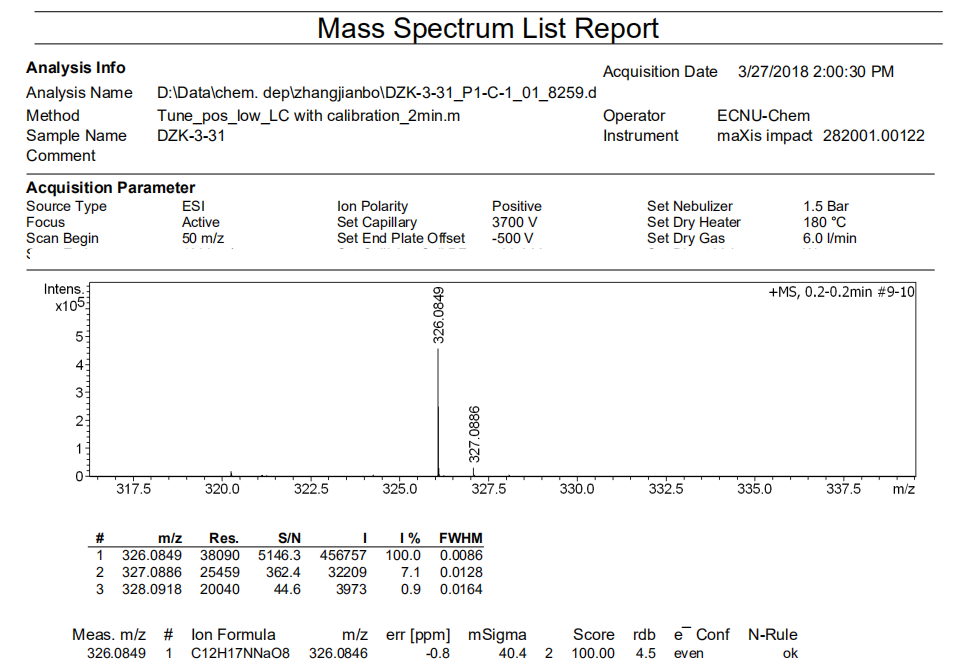
**

**3b**

**
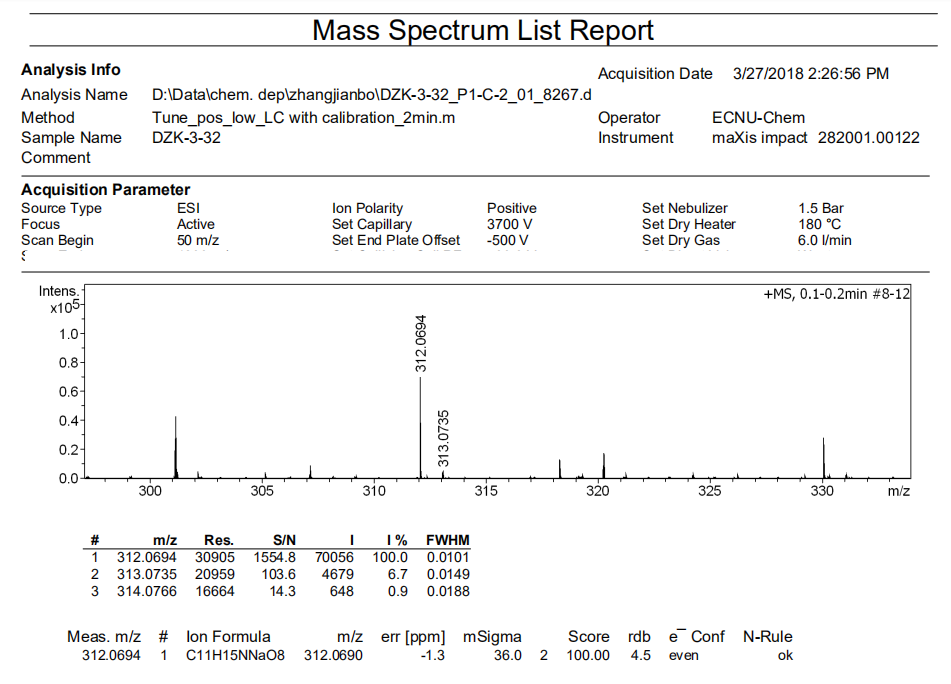
**

**3c**


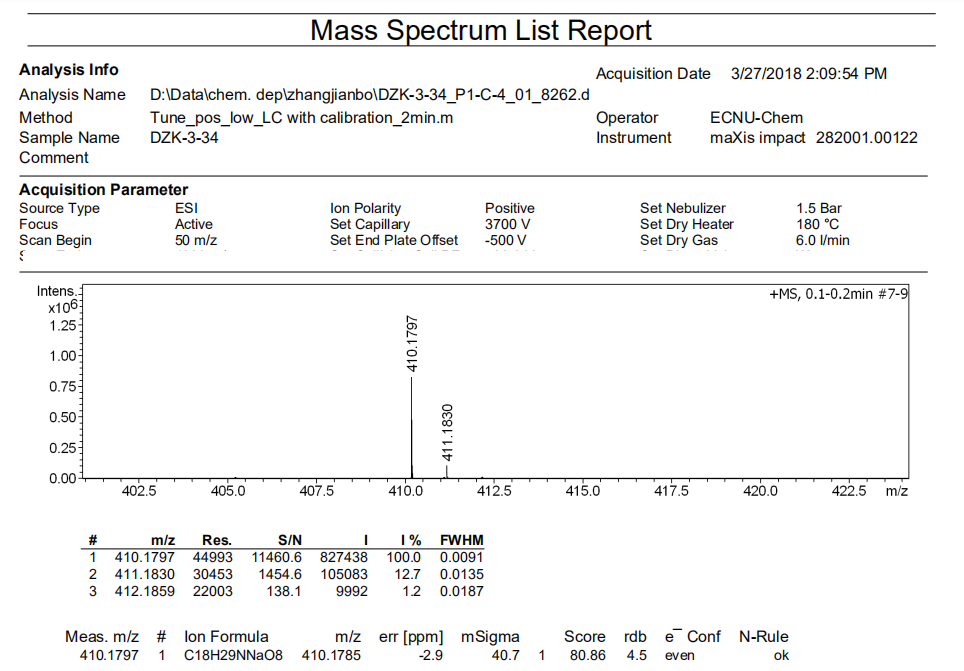


**3d**


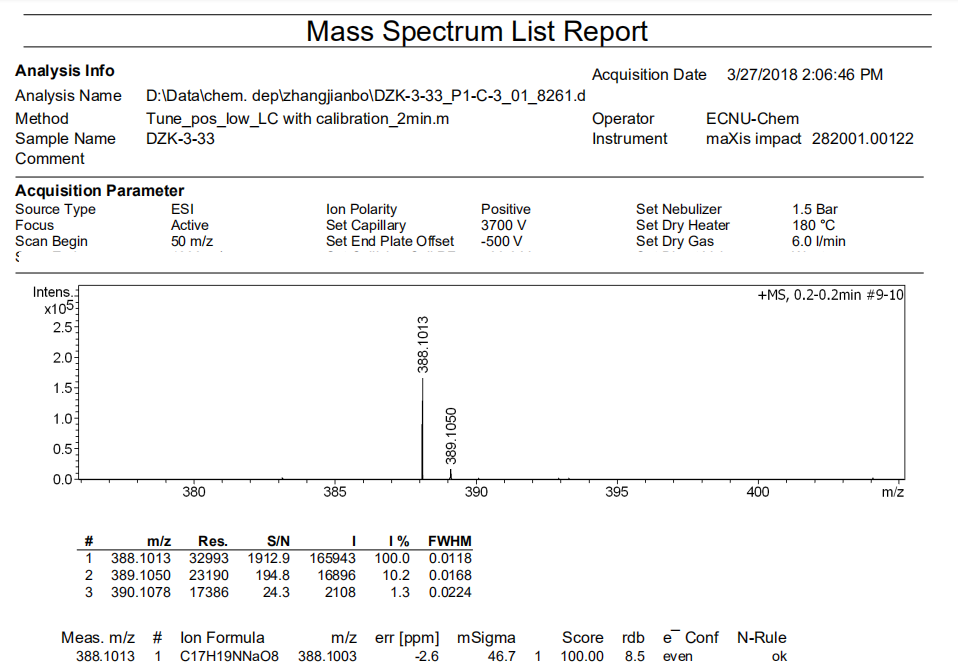


**3e**


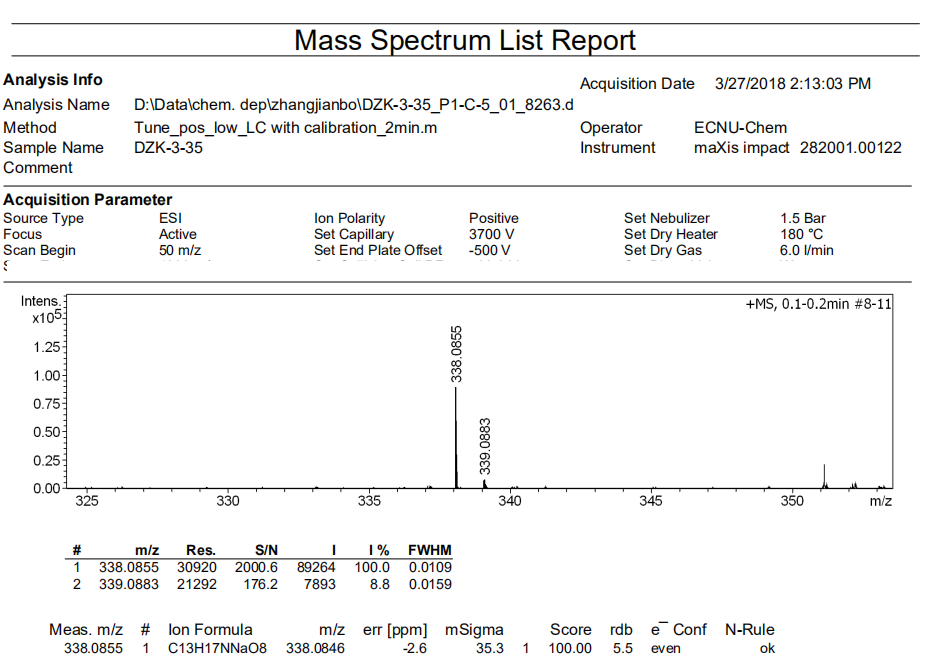


**3f**


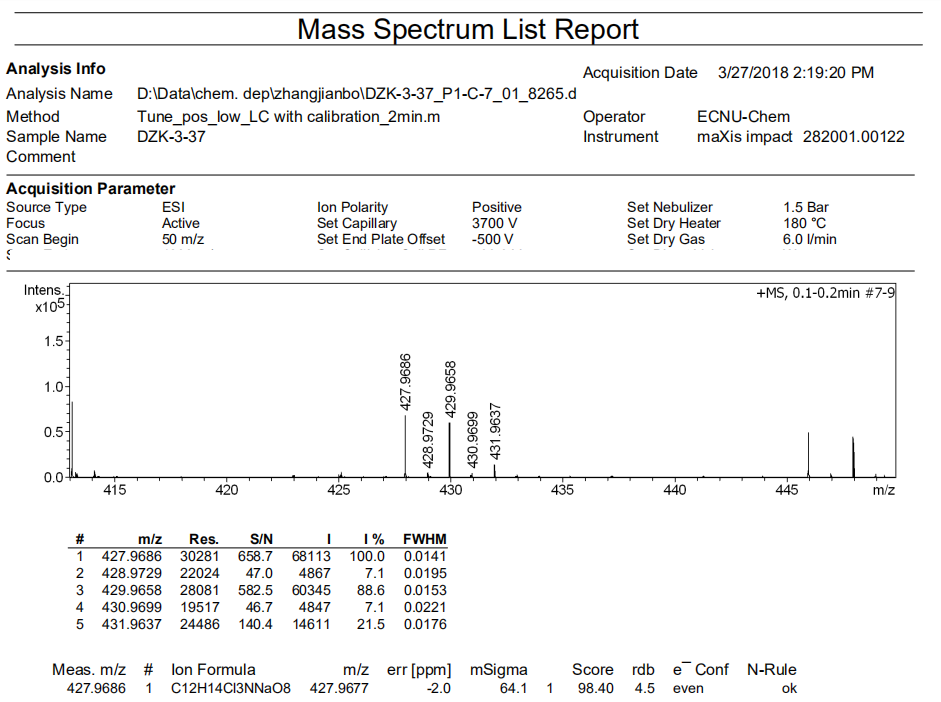


**3g**


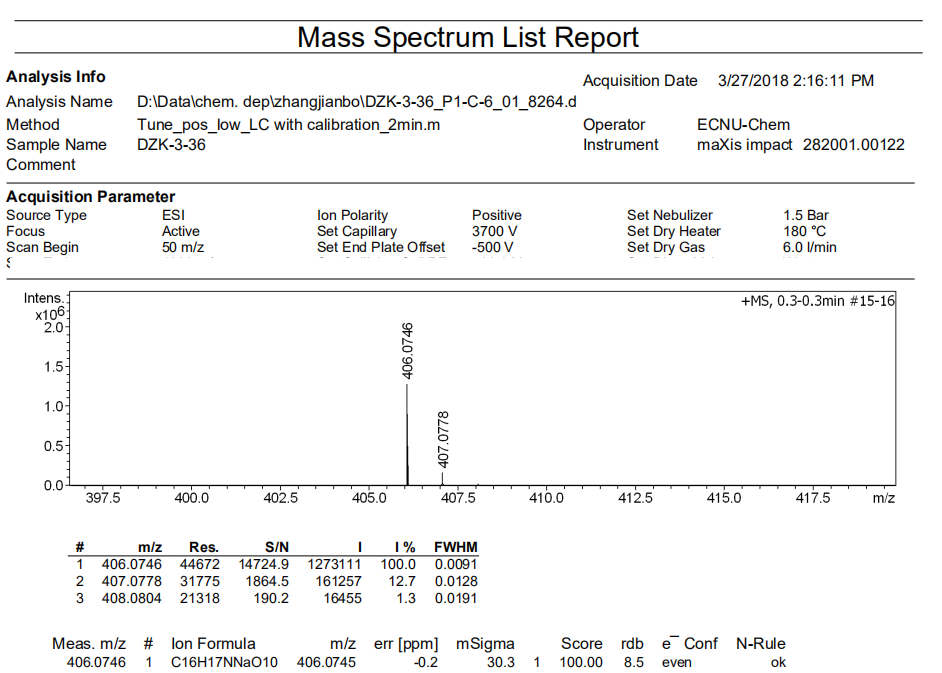


**3h**


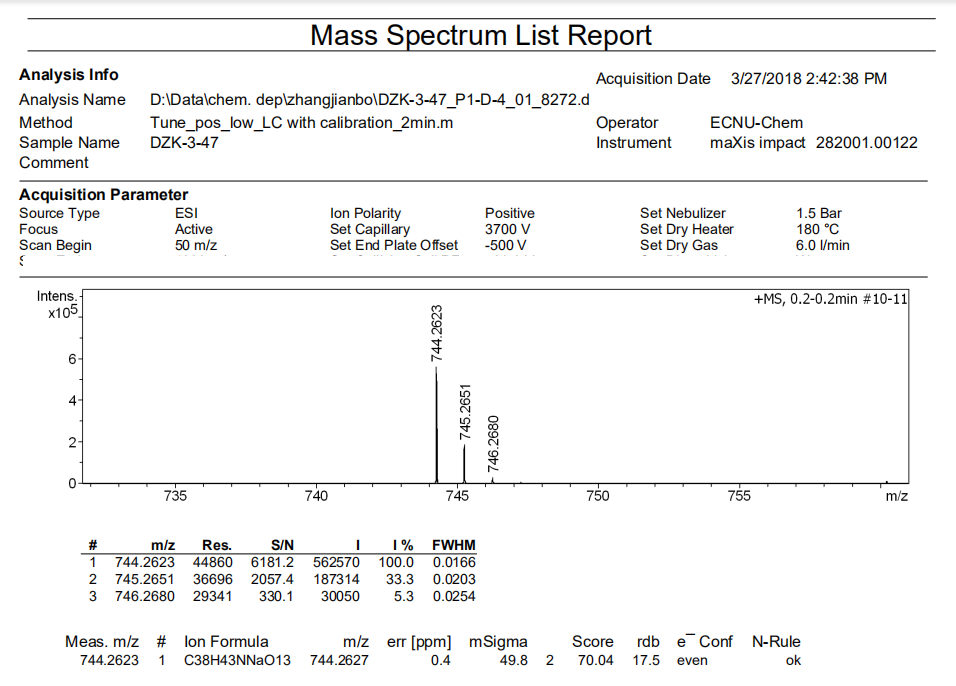


**3i**


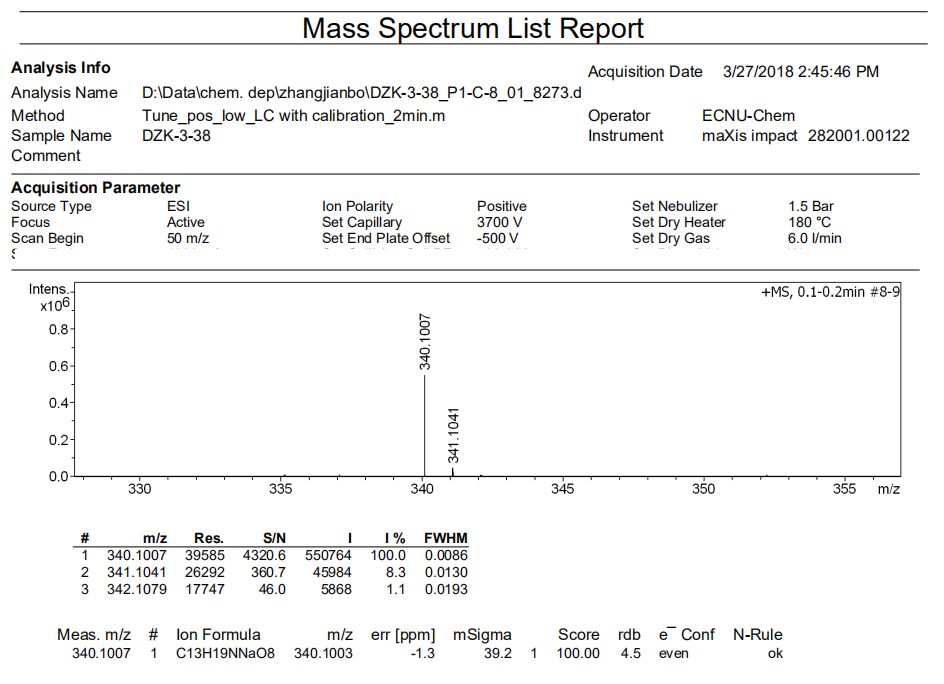
**3j**


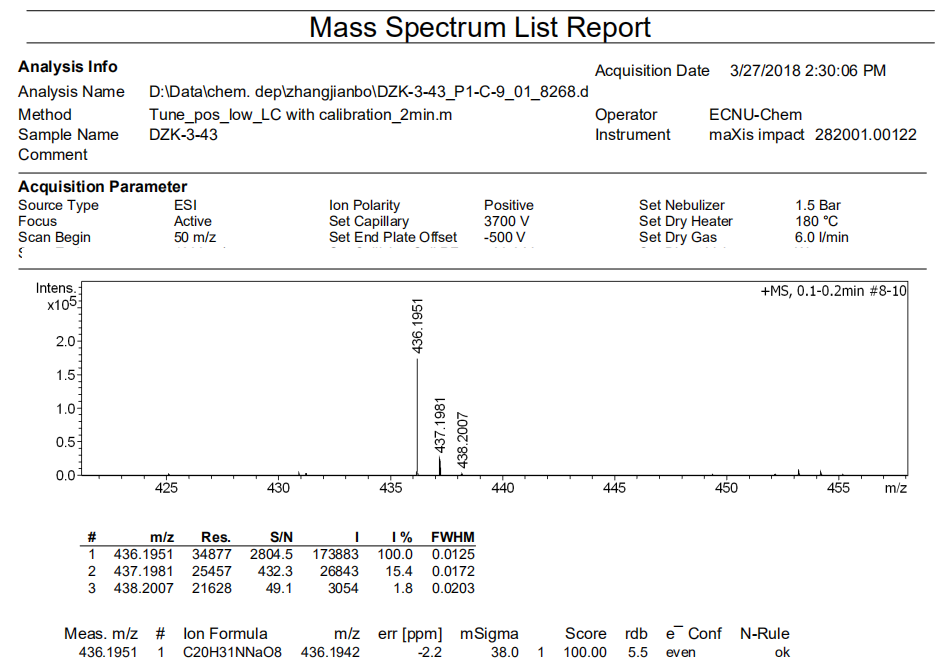


**3k**


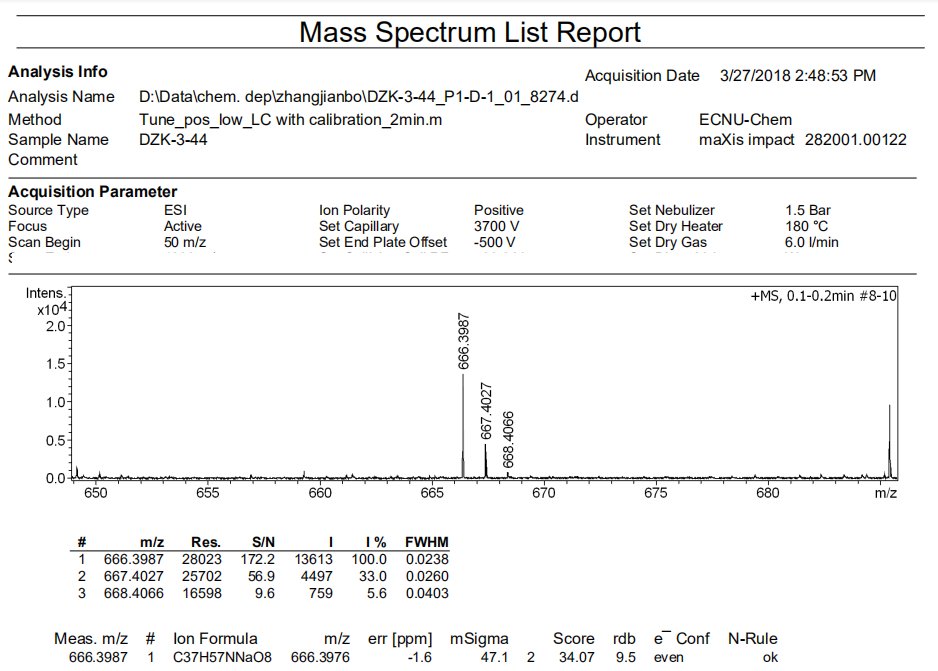


**3l**


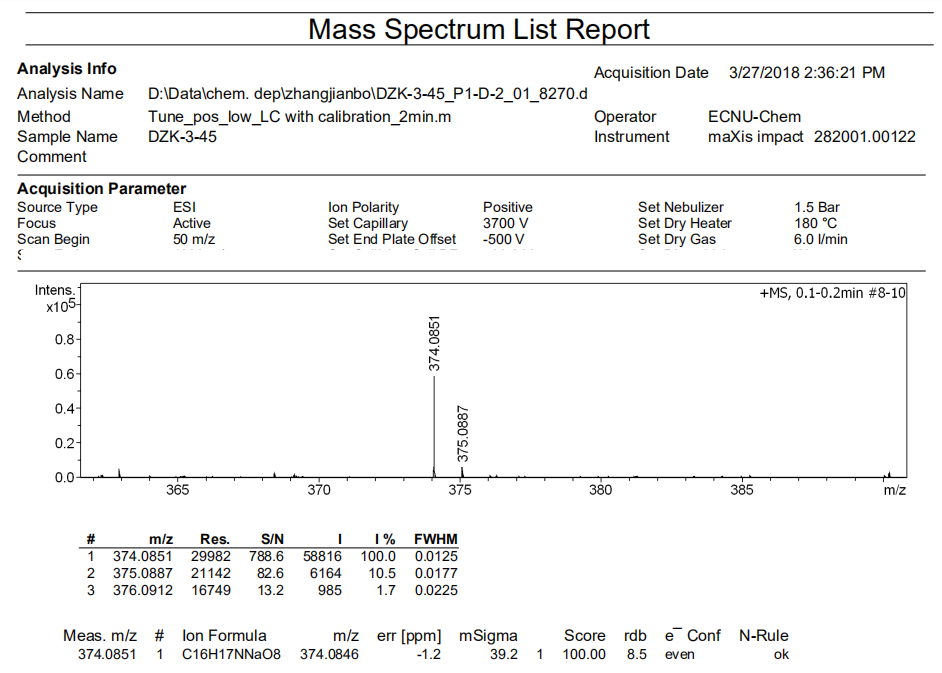


**3m**


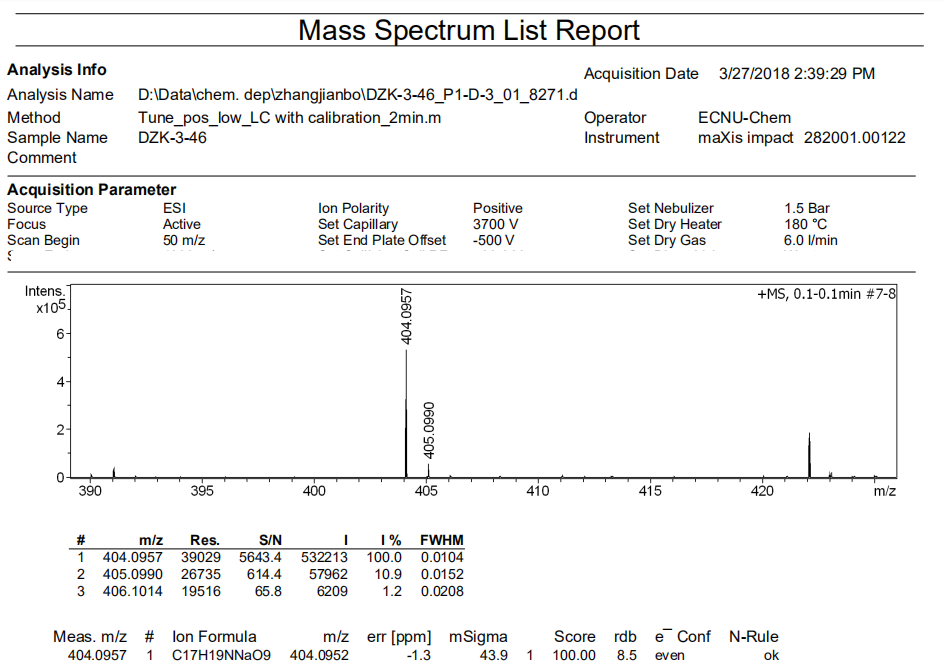

Supplement: Supplementary file 1 [file DataSheet1.docx]
